# Establishment of a novel hepatitis B virus culture system using immortalized human hepatocytes

**DOI:** 10.1038/s41598-020-78655-x

**Published:** 2020-12-10

**Authors:** Yuichi Akahori, Hiroki Kato, Takashi Fujita, Kohji Moriishi, Yasuhito Tanaka, Koichi Watashi, Michio Imamura, Kazuaki Chayama, Takaji Wakita, Makoto Hijikata

**Affiliations:** 1 Institute for Frontier Life and Medical Sciences, Kyoto University, Kyoto, Japan; 2 Graduate School of Biostudies, Kyoto University, Kyoto, Japan; 3 Interdisciplinary Graduate School of Medicine and Engineering, University of Yamanashi, Kofu, Japan; 4 Graduate School of Medical Sciences, Nagoya City University, Nagoya, Japan; 5 Department of Virology II, National Institute of Infectious Diseases, Tokyo, Japan; 6 Department of Gastroenterology and Metabolism, Hiroshima University Hospital, Hiroshima, Japan

**Keywords:** Hepatitis B, Hepatitis B virus

## Abstract

Recent development of hepatitis B virus (HBV) culture systems has made it possible to analyze the almost all steps of the viral life cycle. However, the reproducibility of interaction between HBV and host cells seemed inaccurate in those systems because of utilization of cancer cell lines with a difference from hepatocytes in the majority of cases. In this study, in order to resolve this point, a novel HBV culture system using non-cancer-derived immortalized human hepatocytes derived cell lines, producing exogenous human sodium taurocholate cotransporting polypeptide, was developed. One of the cell clones, E/NtG8 cells, was permissive to both blood-borne HBV (HBVbb) and culture-derived recombinant HBV when cultured in the three-dimensional condition. Furthermore, the production of infectious HBV particles, which showed the similar physicochemical properties to HBVbb, was observed for about a month after HBVbb infection in this system, suggesting that it may reproduce whole steps of the HBV lifecycle under the condition analogous to human liver cells infected with HBV. This system seemed to contribute not only to find novel interactions between HBV and host cells but also to understand mechanism of HBV pathogenesis.

## Introduction

Hepatitis B virus (HBV) is one of the causative agents of acute, fulminant, and chronic hepatitis, liver cirrhosis, and hepatocellular carcinoma (HCC) in the world^1^. In recent years, pegylated interferon-alpha, an immune modulator with antiviral capacity, and the more potent nucleos(t)ide analogs (NAs) have been used for the treatment of chronic HBV infections. One of the NAs, entecavir (ETV), effectively suppresses HBV genome replication in the cytoplasm by inhibiting the priming step, reverse transcription, and DNA-dependent DNA synthesis by HBV polymerase, thereby eliminating HBV from the serum of the patient^2^. However, the cessation of treatment with NA causes the reappearance of HBV in serum, as the covalently closed circular DNA (cccDNA) form of HBV genome is present in the nucleus even in this situation^3^. Prolonged treatment with NA sometimes results in the production of drug-resistant viruses. Furthermore, only a little has been known about the interaction between HBV and the host cells and the pathogenesis of HCC related with HBV infection. In order to study and overcome those problems, the development of new HBV culture system reproducing HBV proliferation in the patients to the extent possible has been long-coveted.

Human sodium taurocholate cotransporting polypeptide (NTCP) is reportedly an HBV entry receptor^4^. The ectopic expression of NTCP would make the cancer-derived cell lines, HuH-7 cells and HepG2 cells, in which the permissiveness to HBV infection has not been observed, susceptible to HBV infection. On the basis of this observation, several cells constitutively producing NTCP have been established and these cells have shown to reproduce all steps of HBV proliferation^4–8^. One of these, the HepG2-hNTCP-C4 cells, were used for identifying several chemical compounds that blocked HBV entry^9–12^. However, these in vitro HBV culture systems have certain limitations for the study of interactions between HBV and cells under physiological conditions possibly because characteristic features of human liver cancer cell lines differ from those of the hepatocytes in human liver^13,14^. For example, efficient production of infectious HBV particles has not been observed in those systems after HBV infection. Moreover, hepatic cancer cells show abnormal innate immune responses^15–18^. Primary human hepatocytes have been utilized for HBV culture systems as a model reflecting the majority of physiological conditions^19,20^. However, the main drawbacks of these culture systems include the brief culture durations in some cases, high cost, and the limited availability for detailed mechanistic analysis. Therefore, for studying the physiological interactions between HBV and the host cells and the production of HBV particles, HBV culture systems using non-cancer-derived cells that are applicable for molecular, biological, and pathogenesis studies are required. It has been reported that the immortalized human hepatocytes, HuS-E/2 cells treated with 2% DMSO for twelve days became susceptible to HBV infection albeit with low efficiency^21^. HuS-E/2 cells have also been shown to support life cycle of blood-borne hepatitis C virus (HCVbb) to some extent^22^. Recent findings of NTCP, and the low level expression of NTCP gene in HuS-E/2 cells suggested the possibility that the high level production of NTCP in HuS-E/2 cells might improve the potential of this cell clone to support HBV life cycle. In this study, therefore, it was intended to develop a novel HBV culture system using HuS-E/2 cells, ectopically producing NTCP.

## Results

### Establishment of immortalized human hepatocytes stably producing NTCP and the verification of the permissiveness to HBV infection

**Figure 1:**
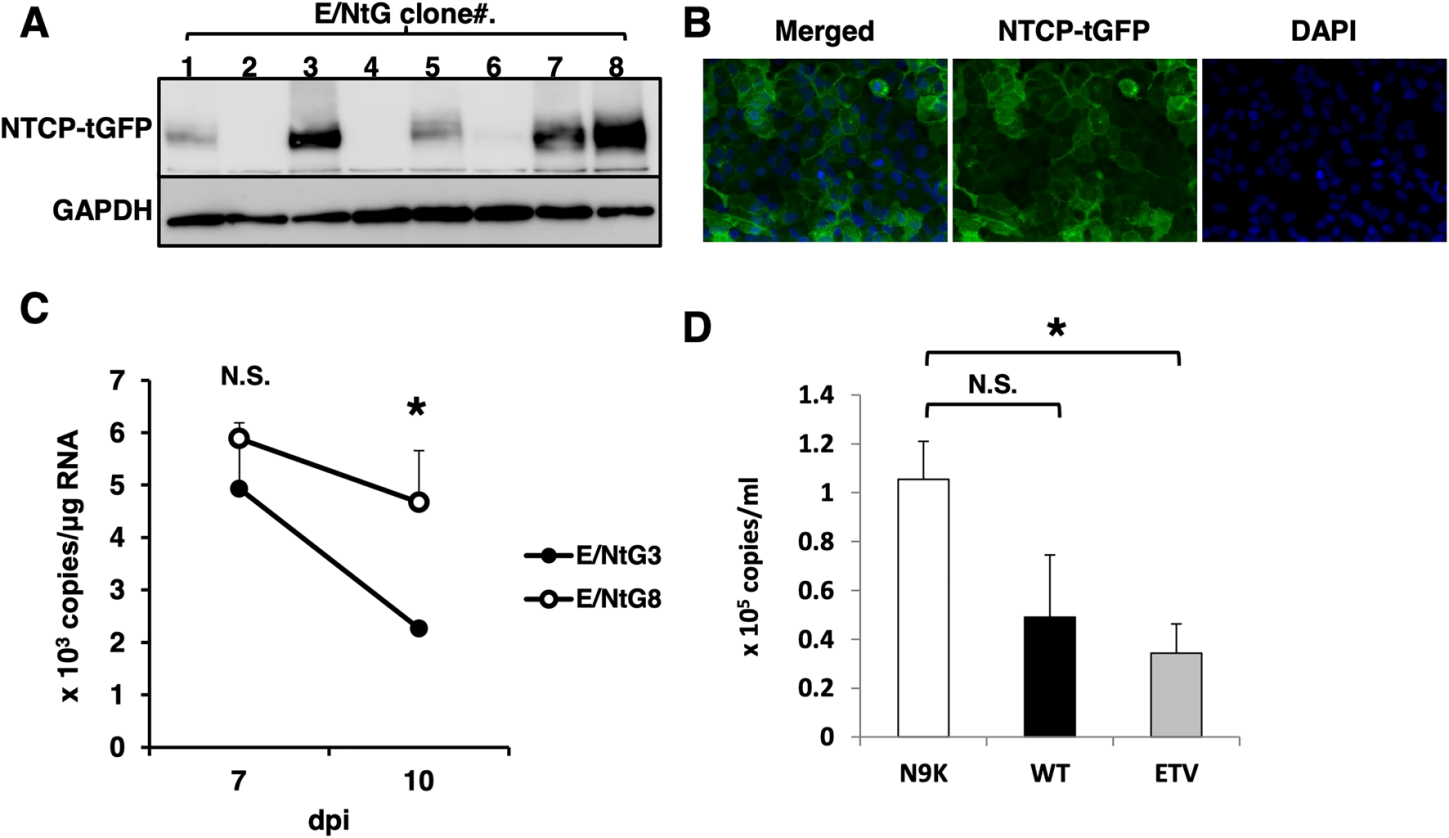
(**A**) Production of NTCP-tGFP was observed by immunoblot analysis using anti-tGFP antibody. GAPDH in the samples was detected by anti-GAPDH antibody to show as the loading control. Full-length blot images were presented in Supplemental Figs. Fig. S8 and Fig. S9. (**B**) Fluorescence imaging of E/NtG8 cells cultured on a collagen-coated chamber slide. NTCP-tGFP and DAPI-stained nuclei were shown in green and in blue, respectively. (**C**) E/NtG3 (closed circle) and E/NtG8 cells (open circle) were infected with HBVcc. The copy number of HBV pgRNA in the cells harvested at the indicated time points was evaluated by qRT-PCR. The dpi indicates “days post infection”. (**D**) E/NtG8 cells were infected with HBVcc in the presence of myr-47WT (WT) (black bar) or myr-47N9K (N9K) (white bar). After 2 weeks of culture in the presence (gray bar) or absence of entecavir (ETV), the extracellular HBV DNA level was evaluated by qPCR. Levels of significance: *p < 0.05. N. S. indicates “no significant difference”.

**Figure 2:**
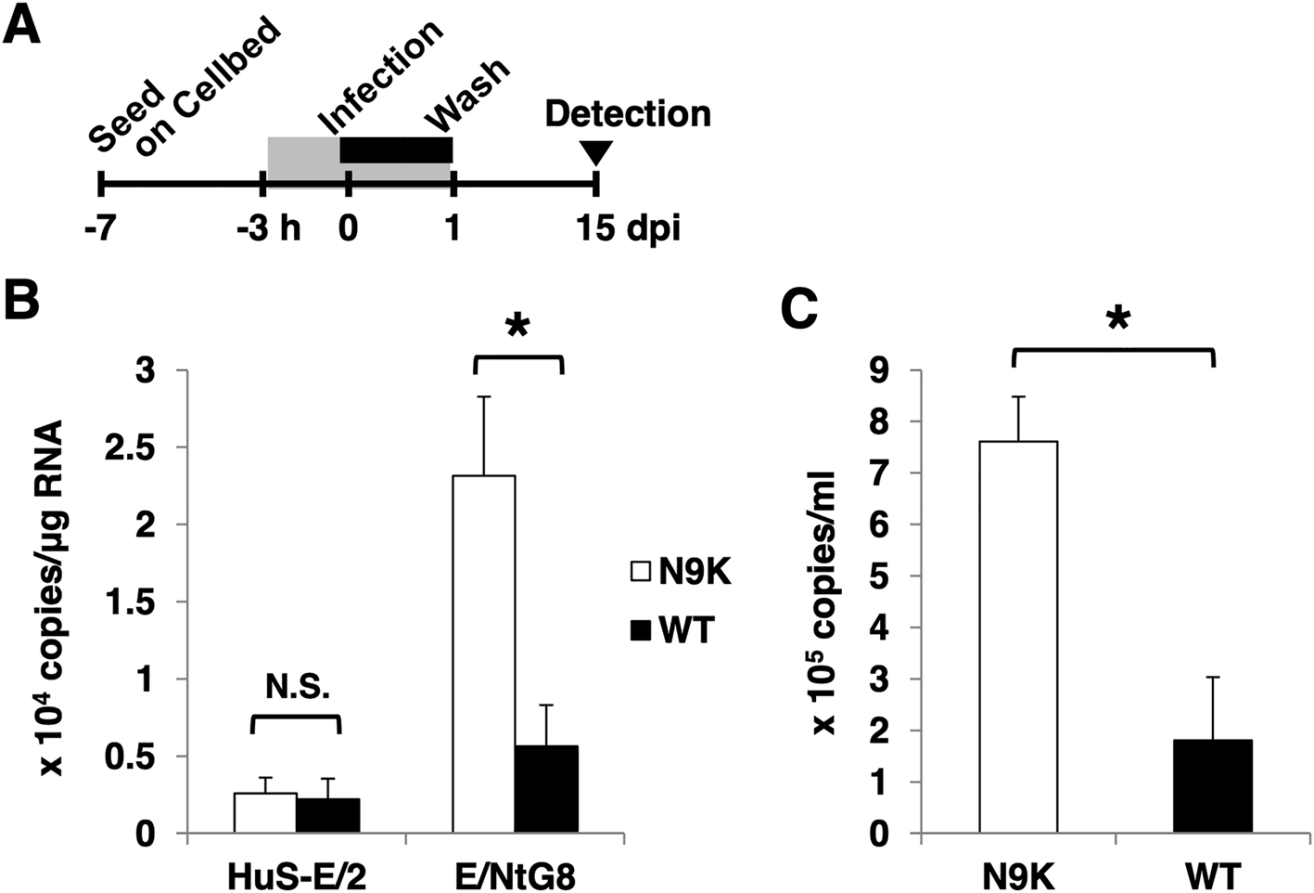
(**A**) Schema of the schedule for HBVcc infection experiment using HuS-E/2 cells and E/NtG8 cells cultured on Cellbed is shown. Inoculation time of the 3D-cultured cells with HBVcc is shown in the black box. The time of treatment with HBV preS1 peptide is shown in the gray box. After infection, the cells were cultured for 2 weeks. (**B**) The amount of HBV pgRNA in 3D-cultured HuS-E/2 cells and E/NtG8 cells infected with HBVcc were evaluated by qRT-PCR at 15 dpi. Data of the cells treated with myr-47WT (WT) or myr-47N9K (N9K) during the inoculation are shown as the following panels. (**C**) The culture media of the HBVcc-infected 3D-cultured E/NtG8 cells were collected every 2 days. The amounts of extracellular HBV DNA in the media were evaluated by qPCR at 15 dpi. Levels of significance: * and N. S. indicate “p < 0.05” and “no significant difference”, respectively.

After selection with G418, several G418-resistant clones were isolated as independent colonies from HuS-E/2 cells transfected with RG210241. Next, eight clones among the G418-resistant clones were selected as candidates according to the expression level of NTCP-tGFP mRNA and named as E/NtG1 to E/NtG8 cells. Among these clones, E/NtG3 and E/NtG8 cells were selected as HuS-E/2-derived cell clones that were high producers of NTCP-tGFP proteins (Fig. 1A). Localization of NTCP-tGFP around the region of the plasma membranes in the cells was observed as expected (Fig. 1B). Next, the permissiveness of each cell clone to HBVcc infection was examined. As shown in Fig. 1C, the copy numbers of HBV pgRNA in E/NtG8 cells appeared to be maintained relatively from 7 to 10 dpi, compared with E/NtG3 cells in which the obvious decrease of HBV pgRNA was detected over the same period. The level of HBV pgRNA expression in E/NtG8 cells (approximately 5 × 10^3^ copies/μg total RNA), however, was lower than that in the other existing HBV culture systems^4^ (see Fig. 2C, approximately 10^4^ to 10^5^ copies/μg total RNA). In addition, the extracellular HBV DNA production from HBVcc-infected E/NtG8 cells was marginal (Fig. 1D). These results suggested that E/NtG3 cells are not permissive to HBV infection, but E/NtG8 cells possess the potential to support HBV infection to some extent with limited ability. The possible contribution of a cellular restriction factor of HBV, SMC6^23^, to the low efficiency of HBV infection in E/NtG8 cells was examined by SMC6 knockdown experiment next. As shown in Supplemental Fig. 1C, the obvious increase of HBV pgRNA was not observed in those cells after HBVcc infection despite effective knockdown of SMC6 mRNA (Supplemental Fig. S1D). As the suppressive effect of SMC6 on the expression of HBV pgRNA was observed in HepG2-NTCP-C4 cells (Supplemental Fig. S1A,B) as previously reported^23^, the low level expression of pgRNA observed in E/NtG8 cells after HBVcc infection was not due to the effect of SMC6 (Supplemental Fig. S1C).

### HBVcc infection in 3D-cultured E/NtG8 cells

Next, the possible improvement of HBV infection in E/NtG8 cells cultured in 3D condition was examined, because parental HuS-E/2 cells have already shown to support the infection and proliferation of HCVbb under the 3D culture condition^24^. In the present study, Cellbed scaffold was utilized for the 3D culture because it was likely to be a better system for the study of virus infections in vitro because cells can be cultured simply and easily in 3D conditions and the cultured cells are directly exposed to the culture medium containing virus particles. Fifteen days after infection with HBVcc, the higher copy number of HBV pgRNA was detected in the 3D-cultured cells treated with myr-47N9K than in the cells treated with myr-47WT (Fig. 2B), while the parental HuS-E/2 cells cultured similarly did not show significant difference (Fig. 2B). The statistical significance of this data suggested that 3D-cultured E/NtG8 cells are permissive to HBVcc infection, unlike 3D-cultured HuS-E/2 cells. Although the level of HBV pgRNA expression in the cells (approximately 5 × 10^3^ copies/μg total RNA) was not much higher than that of the cells in the flat culture condition (Fig. 1C), the noticeable production of extracellular HBV DNA from 3D-cultured E/NtG8 cells was observed at 15 dpi (Fig. 2C). These results suggested that 3D-cultured E/NtG8 cells could support almost all the steps of HBV life cycle.

### HBVbb infection in 3D-cultured E/NtG8 cells

**Figure 3:**
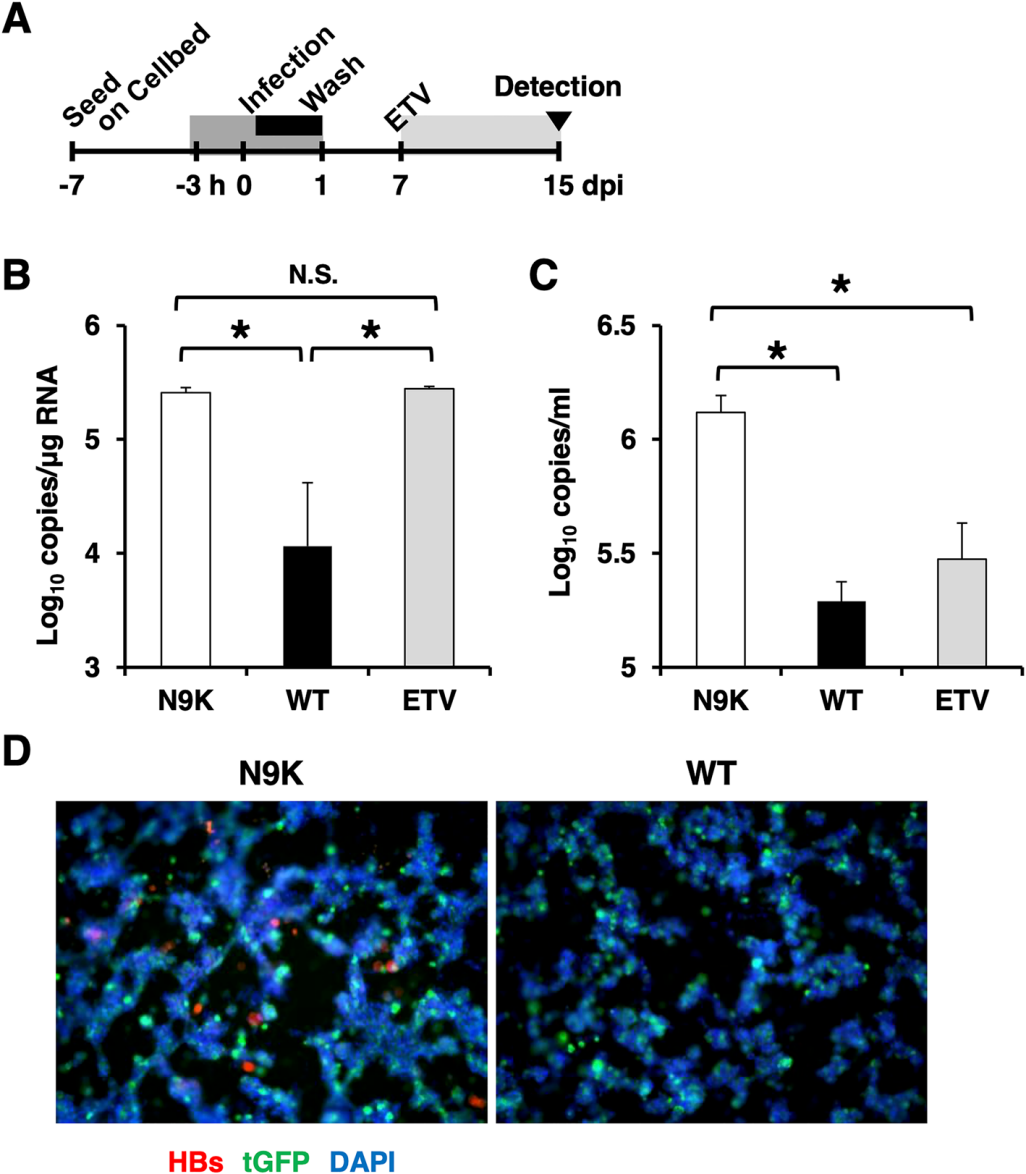
(**A**) Schema of the schedule for HBVbb infection experiment using E/NtG8 cells cultured on Cellbed is shown. Inoculation time of the 3D-cultured cells with HBVbb is shown in the black box. The time of treatment with HBV preS1 peptide is shown in the dark gray box and treatment with ETV is shown in the light gray box. After infection, the cells were cultured for 2 weeks. (**B**) The amount of HBV pgRNA in 3D-cultured E/NtG8 cells infected with HBVbb (genotype C) was evaluated by qRT-PCR at 15 dpi. (**C**) Culture media of the HBVbb (genotype C)-infected cells were collected every 2 days. The amounts of extracellular HBV DNA in the media were evaluated by qPCR at 15 dpi. (**D**) HBVbb (genotype C)-infected cells were immunostained with anti-HBs antibody. The image contains the overlay of HBs (shown in red), NTCP-tGFP (shown in green) and DAPI-stained nuclei (shown in blue). Levels of significance: * and N. S. indicate “p < 0.05” and “no significant difference”, respectively.

Next, the permissiveness of 3D-cultured E/NtG8 cells to HBVbb was examined in the same way. In the case of infection with HBVbb genotype C, HBV pgRNA (2.5 × 10^5^ copies/µg total RNA) in myr-47N9K-treated cells showed a significantly higher copy number than that in myr-47WT-treated cells at 15 dpi without the effect of ETV treatment (Fig. 3B). The formation of HBV cccDNA in HBVbb-infected 3D-cultured E/NtG8 at 15 dpi was also detected (Supplemental Fig. S2). The copy numbers of extracellular HBV DNA from myr-47N9K-treated cells was higher by tenfold at 15 dpi and reached to approximately 1.4 × 10^6^ copies/ml. ETV treatment of myr-47N9K-treated cells caused a large decrease in the extracellular HBV DNA, indicating that the detection of HBV DNA was HBV genome replication-dependent (Fig. 3C). The infection of HBVbb genotype A was also examined in the same way. At 15 dpi, the amount of extracellular HBV DNA from myr-47N9K-treated cells was detected at higher copy number than in myr-47WT-treated cells as in the case of HBVbb genotype C (Supplemental Fig. S3), suggesting that 3D-cultured E/NtG8 cells can support the infection and proliferation of HBVbb of either genotype. Furthermore, the HBs-positive cells were detected in the cells inoculated with HBVbb genotype C in the presence of myr-47N9K at 15 dpi by indirect immunofluorescence analysis (Fig. 3D), indicating that HBs was actually produced in the cells.

### Production of infectious HBV particles from 3D-cultured E/NtG8 cells infected with HBVbb

**Figure 4:**
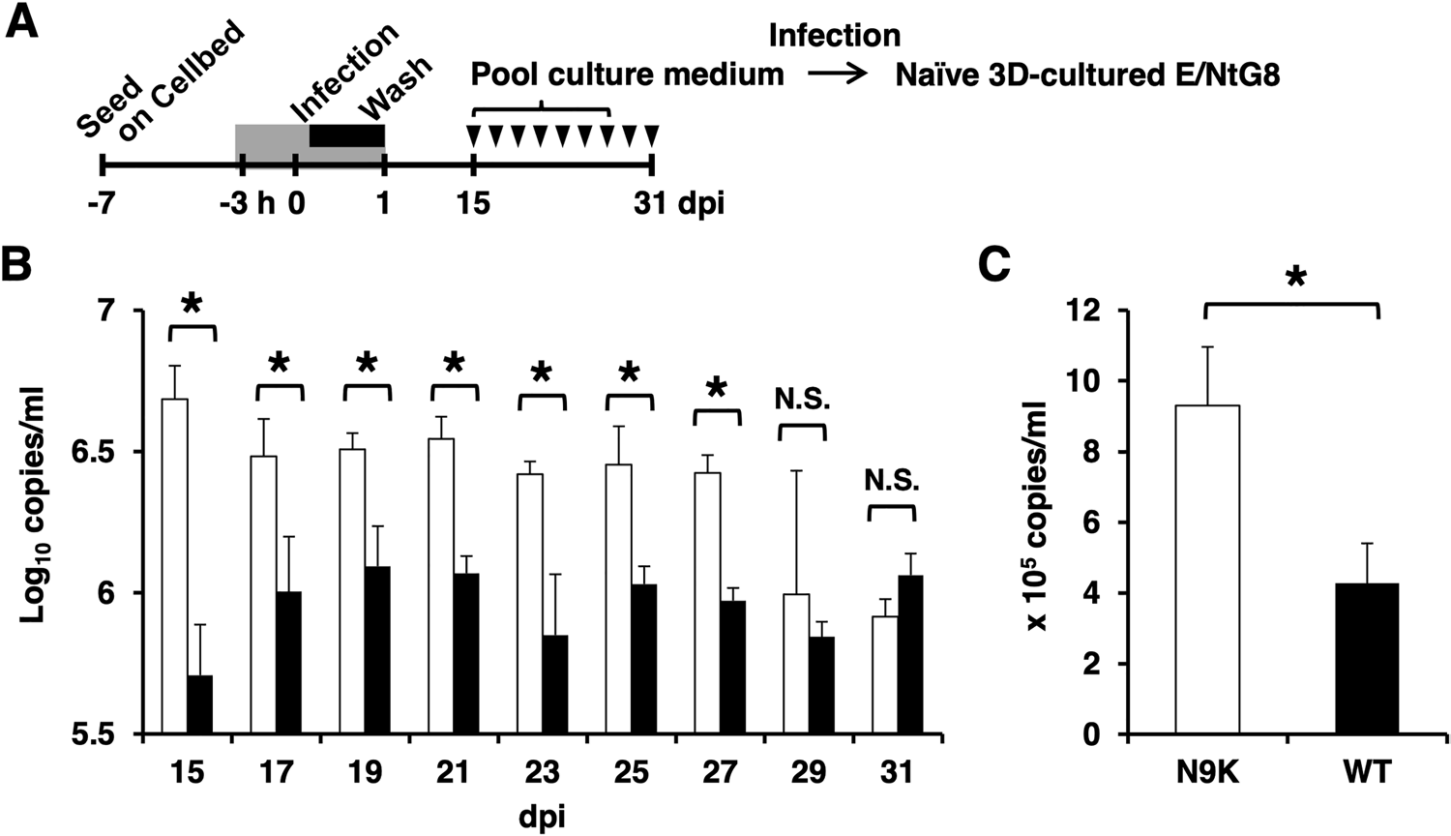
(**A**) Schema of the schedule for HBVbb (genotype C) infection experiment using E/NtG8 cells cultured on Cellbed is shown. Inoculation time of the 3D-cultured cells with HBVbb is shown in the black box. The time of treatment with HBV preS1 peptide is shown in the gray box. After infection, the cells were cultured for 1 month. The culture medium was collected every 2 days (arrowheads) from 15 to 31 dpi. (**B**) The amount of extracellular HBV DNA in each culture medium from 15 to 31 dpi of the cells treated with the inoculum containing HBV preS1 peptide, WT (white bars) or N9K (black bars), was evaluated by qPCR. (**C**) The infection experiment was done as Fig. 3 but using pooled culture medium from 15 to 27 dpi shown in Fig. 4A and 3D-cultured E/NtG8 cells as the infectious source and target cells, respectively. The amount of extracellular HBV DNA at 15 dpi was evaluated by qPCRs. Levels of significance: * and N. S. indicate “p < 0.05” and “no significant difference”, respectively.

As shown in Fig. 4B, the production of extracellular HBV DNA was constantly detected until 27 dpi. The infectious HBV particle production was examined by the infection experiment using pooled culture media as infection source (Fig. 4A). The naïve 3D-cultured E/NtG8 cells were inoculated with the pooled media at 5 HBV GEq/cell as described above. As shown in Fig. 4C, the copy numbers of extracellular HBV DNA from myr-47N9K-treated cells was apparently higher than that from myr-47WT-treated cells at 15 dpi and reached to approximately 10^6^ copies/ml, indicating that the pooled media included the HBV particles which were as infectious as HBVbb. These data suggested that 3D-cultured E/NtG8 cells infected with HBVbb could produce highly infectious HBV particles for about a month. All these data suggested that 3D-cultured E/NtG8 cells could be susceptible to both HBVcc and HBVbb and especially reproduce all the steps of the life cycle of HBVbb including the production of the infectious HBV particles.

### Properties of the HBV particles produced from 3D-cultured E/NtG8 cells

**Figure 5:**
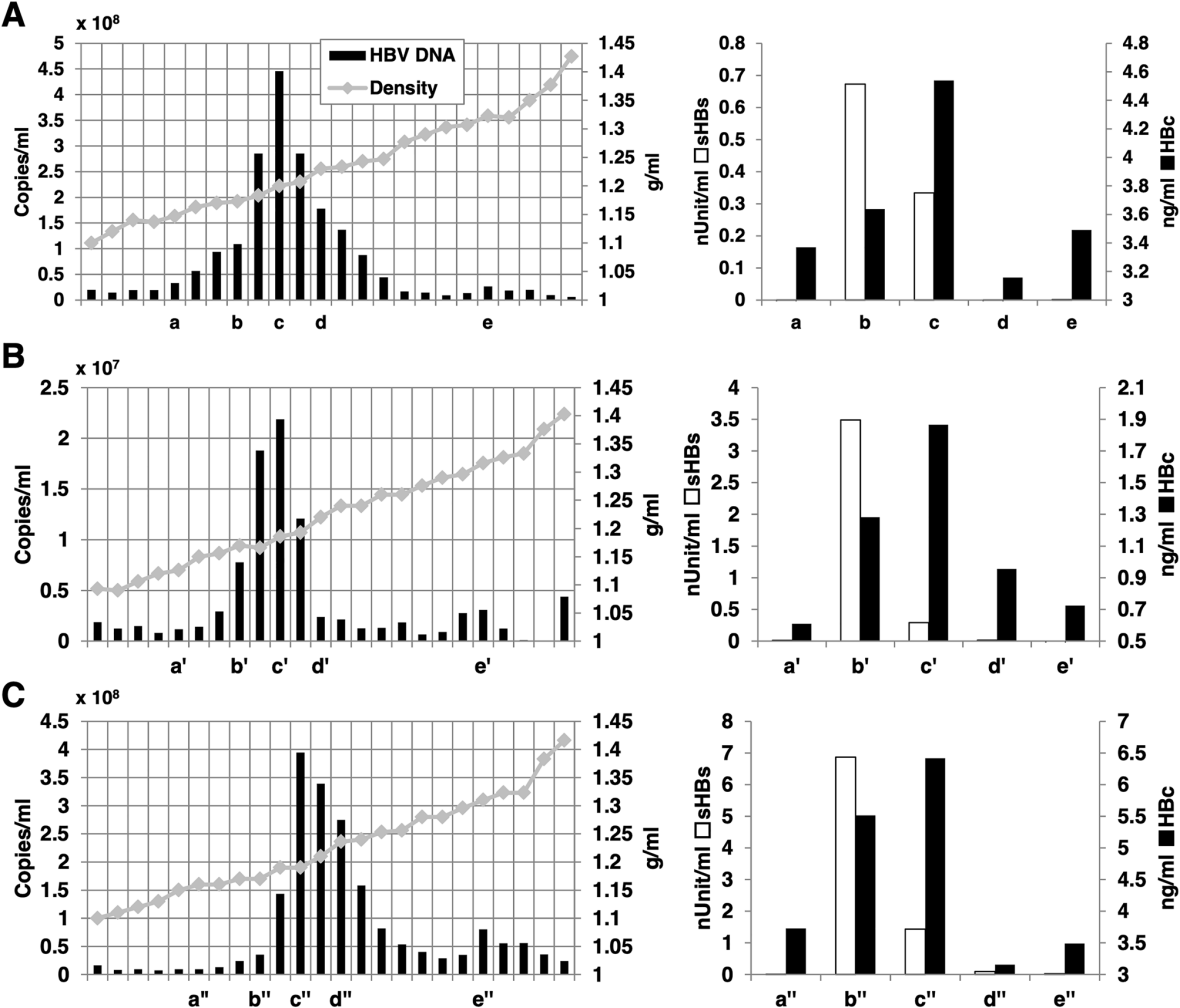
Culture media from 3D-cultured E/NtG8 cells infected with HBVbb (**A**) and HBVcc producing HepG38-7Tet cells (**C**) and serum including HBVbb (**B**) were fractionated by CsCl density gradient ultracentrifugation. For each fraction, the amount of HBV DNA was indicated (black bar) against buoyant density (g/ml, gray dot) (right panels of (**A**–**C**)). The fractions with small letters on horizontal axis were selected for the detection of HBV antigens by ELISA. The amounts of small HBs (SHBs, white bars) and HBc antigens (black bars) in the selected fractions are shown (left panels of (**A**–**C**)).

In the case of infection experiment using PXB cells, the cells only had to be infected with HBVbb on the 5 GEq/cell condition to obtain the assessable and quantitative results (Supplemental Fig. S4A^20^). The infectivity of 3D-cultured E/NtG8-derived HBV, therefore, seemed to be equivalent to the HBVbb as above. On the other hand, when HBVcc was used as an infection source, only a low efficiency of the infection was observed even with the infection on the 8000 GEq/cell condition (Supplemental Fig. S4B). As the infectivity deemed less different among HBV genotypes^20^, it seemed possible to suppose that the inocula of HBV from 3D-cultured E/NtG8 cells and HBVbb include the factor enhancing the HBV infection or that of HBVcc includes a suppressor. However, the presence of those affecting the HBV infection was not observed when the inoculum including both HBVbb and HBVcc was utilized in the HBV infection experiment using PXB cells (Supplemental Fig. S5). Next, therefore, the physicochemical properties of those HBV particles were investigated further by CsCl density gradient ultracentrifugation analysis. Overall distribution patterns of HBV DNA, HBV antigens, small HBs (SHBs) and HBc, and buoyant densities in CsCl densities were similar to those have already reported previously^25^, except the obscure presence of unenveloped core particles. The fractions indicated with c, c’, and c’’ showing the maximum contents of HBV DNA and HBc were likely to contain the infectious HBV particles (Dane particles) (Fig. 5, left panels). And the fractions indicated with b, b’, and b’’ showing the maximum contents of small HBs antigen were presumed to include subviral particles (Fig. 5, right panels). The fractions indicated with e, e’, and e’’ included the minor peak of HBV DNA, probably implying the presence of capsid associated with HBV DNA (Fig. 5). It should be note that those features of 3D-cultured E/NtG8-derived HBV were largely similar to those of HBVbb. Especially the pattern of HBV DNA content showed as a single peak around fractions c and c’. However, that of HBVcc fractions showed the peak with obvious shoulder from fractions c to d (Fig. 5C), although what kinds of particles were included in the fractions corresponding to the shoulder were unknown yet. These results suggested that the physicochemical properties of the HBV particles produced from 3D-cultured E/NtG8 cells bear a resemblance to those of HBVbb, rather than HBVcc. On the other hand, it was observed that the distribution pattern of HBc in each fraction in the right panel of Fig. 5 looked similar between those of panel A and panel C. The difference of the pattens seemed to be caused by higher relative amount of HBc in the fraction e’ than those in fractions e and e’’. In addition, overall distribution pattern of sHBs seemed similar between those of panel B and panel C. However, the contribution of those pattern differences to the infectivity of the viruses was unknown at this moment.

### Candidates of the genes supporting HBV life cycle in 3D-cultured E/NtG8 cells

**Figure 6:**
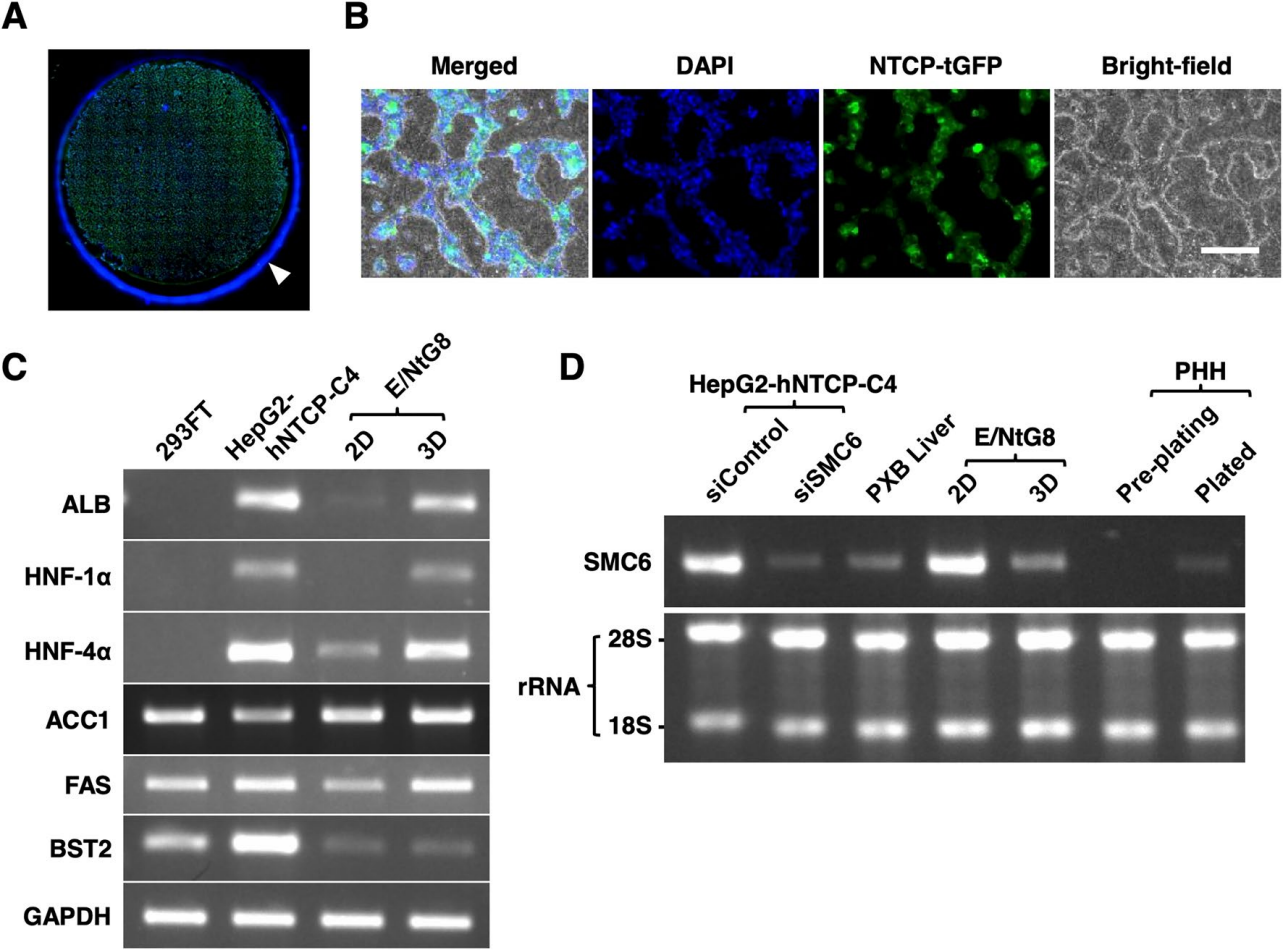
(**A**) Fluorescence imaging of E/NtG8 cells cultured on Cellbed for 7 days is shown. The image contains the overlay of NTCP-tGFP (shown in green) and DAPI-stained nuclei (shown in blue). The diameter of the Cellbed is about 13 mm. The arrowhead indicates a non-specific signal at the edge of the well. (**B**) Higher magnification image of panel (**A**). “Merged” represented the merged images of DAPI/NTCP-tGFP image and bright-field image. White scale bar, 200 µm. (**C**) The mRNA levels of the genes shown on the left of the panel. ALB, HNF-1α, HNF-4 α, ACC1, FAS, and BST-2 in 293FT cells, HepG2-hNTCP-C4 cells, E/NtG8 cells cultured in flat (2D) and 3D-cultured E/NtG8 (3D) cells were semi-quantified by RT-PCR. GAPDH used as an internal control. Full-length gel images were presented in Supplemental Figs. Fig. S10–Fig. S13. (**D**) Expression of SMC6 mRNA in siControl-transfected HepG2-hNTCP-C4, siSMC6-transfected HepG2-hNTCP-C4, PXB mouse liver, 2D-cultured E/NtG8 (2D) and 3D-cultured E/NtG8 (3D) cells, pre-plated cryopreserved human hepatocytes (pre-plating) and human hepatocytes plated and cultured for 48 h (Plated) were evaluated by RT-PCR. 28S and 18S rRNA used as internal and quality controls for each sample of 1 µg total RNA. Full-length gel images were presented in Supplemental Figs. Fig. S14 and Fig. S15.

Our data indicated that the 3D culture condition makes E/NtG8 cells permissive to HBV infection and proliferation. As shown in Fig. 6A,B, the morphology of E/NtG8 cells cultured on Cellbed for one week showed hepatic cord-like structures^26^. In order to obtain the information about cellular factors involved in HBV life cycle in the cells, the expression of several HBV related genes in 3D cultured E/NtG8 cells was investigated as reported previously in the case of HCV^27^. As shown in Fig. 6C, the significantly upregulated expression of the hepatocyte nuclear factor (HNF) family genes, HNF-1α and HNF-4α, which are host transcription factors essential for HBV gene expression^28^, was observed in 3D-cultured E/NtG8 cells, although only low expression levels were detected in the cell cultures in 2D conditions, suggesting that one of the advantages of 3D-cultured E/NtG8 cells would be the high capacity of HBV gene transcription by the increased expression of hepatocyte-specific genes, including HNF family genes. Next, the expression of genes associated with the HBV particle egression, especially genes for fatty acid biosynthesis enzymes^29^, was investigated. Figure 6C shows that acyl-CoA carboxylase (ACC1) and fatty acid synthase (FAS) genes showed increased expression in 3D-cultured E/NtG8 cells, although the increase in ACC1 gene expression seemed rather limited.

Next, the expression of the genes for the factors reported to have anti-HBV potential was also investigated. The expression of the gene for BST-2/tetherin, that was reported to inhibit the release of HBV particles^30^, was significantly lower in E/NtG8 cells cultured in both 2D- and 3D-culture conditions than in HepG2-NTCP-C4 cells (Fig. 6C). The expression of the SMC6 gene in 3D-cultured E/NtG8 cells was also analyzed as described above. The amount of SMC6 mRNA in 2D-cultured E/NtG8 cells was similar to that in HepG2-NTCP-C4 cells treated with control siRNA (Fig. 6D). However, this expression was significantly decreased in 3D-cultured E/NtG8 cells just like that in SMC6 siRNA-transfected HepG2-NTCP-C4 cells (Fig. 6D), in which HBV pgRNA expression was largely enhanced after HBVcc infection (Supplemental Fig. 1A). This data indicated that 3D culture affects SMC6 gene expression in E/NtG8 cells. As observed that the 3D-cultured E/NtG8 cells became more hepatocyte-like cells (Fig. 6A–C), the SMC6 gene expression was examined in several other cells or tissue that share similar features with normal human hepatocytes. As shown in Fig. 6D, compared with HepG2-NTCP-C4 cells and E/NtG8 cells cultured in the 2D culture condition, only low-level expression of SMC6 mRNA was observed in PXB mouse liver and cryopreserved human hepatocytes cultured for 48 h. Furthermore, SMC6 mRNA was less expressed in cryopreserved human hepatocytes before culturing on the culture plate. These results suggest that SMC6 gene expression is limited in human hepatocytes in the liver tissue, although the functional potential of the protein in the hepatocytes of human liver remains unclear. It was appeared that the expression of genes related with HBV lifecycle was regulated in the E/NtG8 cells in 3D-culture conditions to support HBV proliferation.

## Discussion

Numerous HBV culture systems have been reported and used for HBV research. Liver cancer-derived cells such as HepG2, HuH-7, and HepaRG cells have been commonly used in these systems, while primary human hepatocytes and PXB cells have also utilized in some cases^31,32^. Hepatocyte-like cells developed by differentiation from induced pulmonary stem (iPS) cells for the reproduction of HBV infection and proliferation have been previously reported^33–35^. Each system mentioned above has both merits and demerits. In the case of systems using cancer-derived cells, especially recently developed cell lines constitutively producing NTCP, almost the entire HBV life cycle can be easily studied. Selected cell clones from those cells recently showed better reproduction of the HBV life cycle^36,37^. Nevertheless, the investigation of the interactions between the host cell and HBV under physiological condition seems to be limited because of the abnormalities in cancer-derived cells. The iPS-derived hepatocyte-like cells may be one of the most ideal systems to study this subject, but the efficacy of HBVcc infection is quite low at this moment. Those cells also seemed to require investigation for their potential to produce infectious HBV particles and if they can be highly permissive to HBVbb infection such as PXB cells and 3D-cultured E/NtG8 cells, although the exact differences between HBVcc and HBVbb are not yet known.

Because HuS-E/2 cells, the parental cells of E/NtG8 cells, have already shown to support the infection and proliferation of HCVbb through becoming matured to hepatocyte-like cells in 3D culture conditions^22,24,27^, it seemed possible that the 3D culture conditions improve cellular environment of E/NtG8 cells for HBV proliferation as well. Then the expression profiles of several genes, which contribute to HBV lifecycle, in 3D-cultured E/NtG8 cells were investigated. The expression levels of the genes for hepatocyte-specific transcription factors such as HNF-1α and HNF-4α that are known as regulators for HBV gene expression^38,39^ were induced in 3D-cultured E/NtG8 cells (Fig. 6C). It is likely that the relatively high level expression of those factors are required for the adequate transcription of HBV genes from HBV cccDNA as well as host genes supporting HBV infection and proliferation. In this study, the production of infectious HBV particles that have similar characteristic features to HBVbb from 3D-cultured E/NtG8 cells were also observed (Figs. 3, 5). Since the fatty acid biosynthesis pathway contributes to HBV particle production^29^, the elevated expression levels of ACC1 and FAS genes observed in 3D-cultured E/NtG8 cells suggests the advantage in the production of infectious HBV particles. Although the expression of the genes for several factors of the endosomal sorting complex, that are required for the transport pathway in HBV particle egression, such as α-glycosidase, AAA-type ATPase Vps4, charged multivesicular body protein 3 (CHMP3), and CHMP4^40–42^, were also observed in E/NtG8 cells, an obvious alteration of the gene expression was not found (Supplementary Fig. S6). In addition, the gene expression of an antiviral factor, BST-2/tetherin, which was suggested to inhibit HBV release from the cells^30^, was observed to be low in E/NtG8 cells irrespective of the culture condition. These data of the gene expression levels may partly explain the advantage of 3D-culture conditions for E/NtG8 cells in terms of production of infectious HBV particles.

In this study, some crucial differences were noted between 2D- and 3D-cultured E/NtG8 cells. A recent study suggested that the SMC 5/6 complex functions as a host restriction factor of HBV replication by suppressing transcription from the extrachromosomal DNA such as HBV cccDNA^23^. This suppressive function of SMC6 was confirmed in HBVcc-infected HepG2-NTCP-C4 cells by decreased expression of this factor by siRNA, although a significant effect was not observed in HBVcc-infected 2D-cultured E/NtG8 cells, which could be attributed to the limited expression levels of the essential transcription factors. Therefore, it was expected that the suppression of SMC6 expression improved the efficacy of HBV infection in 3D-cultured E/NtG8 cells producing those factors at a level higher than that produced in 2D-cultured cells. Unexpectedly, however, SMC6 gene expression was downregulated in 3D-cultured E/NtG8 cells. Since the mRNA level of SMC6 in 3D-cultured E/NtG8 cells was largely similar to that in SMC6 knockdown HepG2-NTCP-C4 cells, the suppressive effect of SMC6 on HBV transcription seemed to be limited in 3D-cultured cells, suggesting that one of the advantages of 3D culture could be cancellation of this cellular restriction machinery against HBV. Nevertheless, it seemed possible that SMC6 gene is expressed at marginal levels in the human liver tissue because the apparent expression of the gene was not detected in both cryopreserved primary human hepatocytes and in the liver tissues of a chimeric mouse with human hepatocytes. The detection of SMC6 mRNA in primary human hepatocytes at two days after plating on the culture dish suggested that SMC6 gene expression could be induced by 2D culture conditions. Further investigations are required for understanding the role of SMC6 in HBV infection in the human liver.

In contrast to HepG2-NTCP-C4 cells, 3D-cultured E/NtG8 cells showed much higher permissiveness to HBVbb infection than HBVcc. Under the condition that HBVbb inoculum was used only at 5 GEq/cell, the infection of HBVbb was observed in 3D-cultured E/NtG8 cells as well as PXB cells^20^, but not HepG2-NTCP-C4 cells (Supplemental Fig. S7). Since the detectable infection of HBVcc was similarly observed when both HepG2-NTCP-C4 cells and 3D-cultured E/NtG8 cells were infected with HBVcc at 8,000 GEq/cell, the different susceptibility to HBVbb between HepG2-NTCP-C4 cells and 3D-cultured E/NtG8 cells seemed due to the lack of requirement(s) in HepG2-NTCP-C4 cells to support HBVbb infection. Further analysis of these requirements may provide an insight into understanding HBV infection in physiological conditions. In addition, it will be important to examine that the expression of genes in the host cells is affected during HBVbb or HBVcc infection.

In conclusion, we developed a novel in vitro culture system combining 3D-culture scaffold and NTCP-expressing immortalized hepatocytes and demonstrated the relatively efficient support of HBV lifecycle, including production of highly infectious HBV particles. This system may be of benefit for studying the interactions between HBV and host cells at the molecular level in physiological conditions and for understanding mechanism of HBV pathogenesis.

## Methods

### Antibodies

Rabbit polyclonal anti-turbo green fluorescent protein (tGFP) antibody AB513 was purchased from Evrogen (Moscow, Russia). Mouse monoclonal antibodies against HBV surface protein (HBs) (NCL-HBsAg-2) and glyceraldehyde-3-phosphate dehydrogenase (MAB374) were obtained from Leica (Wetzlar, Germany) and Millipore (MA, USA), respectively.

### Reagents

The myristoylated HBV preS1 peptides, myr-47WT and myr-47N9K^7^, were obtained from Scram (Tokyo, Japan). Structural maintenance of chromosomes protein 6 (SMC6) siRNA and non-specific control siRNA were purchased from Dharmacon (Colorado, USA). Dimethyl sulfoxide (DMSO) was obtained from Nacalai Tesque (Kyoto, Japan).

### Plasmid

RG210241, a mammalian expression vector for NTCP tagged with tGFP on the carboxyl-terminal end (NTCP-tGFP)^43^ was obtained from OriGene Technologies (Maryland, USA).

### Cell culture

HuS-E/2 cells and the cell clones derived from HuS-E/2 cells were cultured using a specialized culture medium for the maintenance of HuS-E/2 cells in the presence and absence of 500 μg/ml G418 (Nacalai Tesque, Kyoto, Japan), respectively, as previously described^22^. Transfection of RG210241 into HuS-E/2 cells was performed using Effectene Transfection Reagent (Qiagen, Hilden, Germany). For 3D cell culture, E/NtG8 cells were cultured with serum-free medium (SFM)(TOYOBO, Osaka, Japan) on Cellbed (the high-purity silica fiber scaffold, Japan Vilene, Tokyo, Japan)^44^ in a 24-well plate (5 × 10^5^ cells per well) for one week before the infection experiment. In the case of 3D culture for HepG2-hNTCP-C4 cells, the cells were similarly cultured on Cellbed except for the cell number (5 × 10^5^ cells per well) and three days of culture before infection experiment. The overlaid culture medium was changed every 2 days. The 293FT cells were cultured as previously described^17^. HepG2.2.15.7 and HepG2-hNTCP-C4 cells were maintained as previously described^9,45^. Cryopreserved human hepatocytes (HHs, HU4224) were purchased from Invitrogen (CA, USA). PXB cells were purchased from PhoenixBio (Hiroshima, Japan).

### Immunoblot analysis

Immunoblot analysis was performed as previously described^46^. The HRP-catalyzed chemiluminescence in the Western Lightning reagent (Perkin Elmer, Massachusetts, USA) was detected using ImageQuant LAS-4000 system (GE Healthcare, Chicago, USA) according to the manufacturer’s protocol.

### Indirect immunofluorescence analysis

Indirect immunofluorescence analysis was performed as previously described^47^. The anti-HBs antibody was used as the primary antibody at 1:200 dilution, and the secondary antibody, Alexa 546-conjugated anti-mouse IgG antibody (Invitrogen, California, USA), was used at 1∶1000 dilution. Nuclei were stained with 4′,6-diamidino-2-phenylindole (DAPI). Fluorescence imaging was performed with AF6000, a fluorescence microscope (Leica, Wetzlar, Germany), or FV1000, a laser scanning confocal microscope (Olympus, Tokyo, Japan).

### HBV preparation and conditions of infection

The infectious recombinant HBV particles (HBVcc, HBV derived from cell cultures) were prepared from a culture medium used for HepG2.2.15.7 cells that permanently produce particles of HBV genotype D as previously described^45^. HBV DNA of HBVcc was quantified by quantitative PCR after treatment with micrococcal nuclease (MNase) (New England Biolabs, MA, USA). Blood-borne HBV, henceforth referred to as HBVbb, was obtained as the sera of chimeric mice with humanized liver infected with HBV genotype C and genotype A (PhoenixBio, Hiroshima, Japan)^20,48^. After the quantification of HBV DNA in the media and sera, HBVcc and HBVbb were used in infection experiments at 8 × 10^3^ genome equivalent (GEq) per cell and 5 GEq/cell, respectively, in the presence of 4% PEG-8000 as previously described^9,20^. Almost all infection experiments about HBVcc and HBVbb were performed with pre- and co-treatments with myristoylated HBV preS1 peptide (myr-47WT) to block the entry of HBV into the cell and the single amino acid mutant of the peptide (myr-47N9K), which lacks inhibitory activity, as shown in Figs. 2A, 3A and 4A^4,7^.

### Total RNA extraction from cultured cells, reverse transcription polymerase chain reaction, and quantitative RT-PCR

Total cellular RNA was isolated from the cells using the RNeasy Plus Mini Kit (Qiagen, Hilden, Germany) after treatment with RNase-free DNase (Qiagen). RT-PCR was performed using One-Step RNA PCR Kit (Takara, Osaka, Japan) with target-specific primer sets (listed in Table 1 with PCR cycles). HBV RNA was quantified using ReverTra Ace qPCR RT Kit (TOYOBO, Osaka, Japan) and Universal SYBR Green Master Mix (Applied Biosystems, CA, USA). All these experiments were performed according to the manufacturers’ protocols.

**Table 1:** The sequence of target-specific primer sets (F, forward primer; R, reverse primer).

GAPDH mRNA	F	5′-GCCGCATCTTCTTTTGCGTC-3′
	R	5′-TCGCCCCACTTGATTTTGGA-3′
ALB mRNA	F	5′-TTGCCTTCATTAGCTGCTGA-3′
	R	5′-TTGCTGCCCACTTTTCCTAGG-3′
HNF-1α mRNA	F	5′-GTGTCTACAACTGGTTTGCC-3′
	R	5′-TGTAGACACTGTCACTAAGG-3′
HNF-4α mRNA	F	5′-TGCGACTCTCCAAAACCCTC-3′
	R	5′-AGCCCGGAAGCATTTCTTGA-3′
SMC6 mRNA	F	5′-TGTTGGCGAAATGAACCGGA-3′
	R	5′-ACTGCACTCTTCCCACTTCC-3′
ACC mRNA	F	5′-CTCTTGGCCTTTTCCCGGTC-3′
	R	5′-GTCTGGTTCATCCACGAGCA-3′
GAA mRNA	F	5′-AGCAGTACCTGGACGTTGTG-3′
	R	5′-TTGAACGTGAAGTCCCTCCG-3′
Vps4A mRNA	F	5′-CCAAGTGCGTGCAGTACCTA-3′
	R	5′-TCCACCGTATGTTGGGCTTC-3′
CHMP3 mRNA	F	5′-CATGCGCAACATCCCCTAGT-3′
	R	5′-GGCTTCTCCTGGGTsCTTTCC-3′
CHMP4 mRNA	F	5′-TCCCACTGACCCCATACACT-3′
	R	5′-CGACATCAAGAGGCGCAAAC-3′
BST2 mRNA	F	5′-TCCCACTGACCCCATACACT-3′
	R	5′-CGACATCAAGAGGCGCAAAC-3′
FAS mRNA	As previously described^29^	
HBV pgRNA	As previously described^4^	
Genotype D-specific primer	F	5′-ATGGGGCAGAATCTTTCCAC-3’
	R	5′-TTGTGGTGTTTGCTCTGAAG-3’

### Extracellular HBV DNA extraction and quantitative PCR

Extracellular HBV DNA was isolated from the culture medium or sera with DNeasy Blood and Tissue kit (Qiagen) following MNase treatment with 10 U per reaction at 37 °C for 1 h, as previously described^29^. MNase-resistant extracellular HBV DNA was quantified by quantitative PCR using THUNDERBIRD Probe qPCR Mix (TOYOBO, Osaka, Japan) with a target-specific TaqMan probe and primer sets as previously described^29,48^. In Supplemental Fig. S5, genotype D-specific HBV DNA was quantified using PowerUp SYBR Green Master Mix (Thermo Fisher Scientific, MA USA) and specific primers (listed in Table 1).

### Short-interfering RNA (siRNA) transfection

HepG2-hNTCP-C4 cells and E/NtG8 cells (1 × 10^5^ cells/well in 12-well collagen-coated plates in both cases) were transfected with 100 nM of SMC6 siRNA (#D-018408-02) and non-specific control siRNA (#D-001210-03-05) (Dharmacon, CO, USA) using Lipofectamine RNAiMAX Reagent (Invitrogen) according to the manufacturer’s protocol.

### Cesium chloride density gradient centrifugation

After concentration as described above, HepG38.7-Tet cell-derived HBVcc and Cellbed culture-derived HBV samples were reconstituted with SFM (1 × 10^8^ copies/sample). HBVbb from HBV infected PXB mouse was diluted with SFM. These samples were fractionated using 5–40% (w/v) CsCl density-gradient centrifugation at 28,000 rpm (P55ST2; Hitachi Koki, Tokyo, Japan) at 4 °C for 16 h. After measurement of the buoyant density, the fractions were used for further analyses following the removal of CsCl by ultrafiltration Microcon YM-30 (Millipore, MA, USA).

### Enzyme-linked immunosorbent assay (ELISA)

Quantitation of small HBs (SHBs) and HBc were performed by HBs S Antigen Quantitative ELISA Kit, Rapid–II (Beacle; Kyoto, Japan) and QuickTiter HBV Core Antigen ELISA Kit (Cell Biolabs, CA, USA), respectively, according to the manufacturer’s protocols.

### Statistical analysis

All experiments were independently performed at least three times. Statistical analyses were performed using Student’s t-test; p < 0.05 was considered statistically significant.

## Supplementary Information

Supplementary Information.

## Data Availability

All data generated or analyzed during this study are included in this published article and its Supplementary Information files.

## References

[1] Liang TJ, Block TM, McMahon BJ, Ghany MG, Urban S, Guo JT, Locarnini S, Zoulim F, Chang KM, Lok AS (2015). Present and future therapies of hepatitis B: from discovery to cure. Hepatology.

[2] Seifer M, Hamatake RK, Colonno RJ, Standring DN (1998). In vitro inhibition of hepadnavirus polymerases by the triphosphates of BMS-200475 and lobucavir. Antimicrob. Agents Chemother..

[3] Chou YC, Jeng KS, Chen ML, Liu HH, Liu TL, Chen YL, Liu YC, Hu CP, Chang C (2005). Evaluation of transcriptional efficiency of hepatitis B virus covalently closed circular DNA by reverse transcription-PCR combined with the restriction enzyme digestion method. J. Virol..

[4] Yan H, Zhong G, Xu G, He W, Jing Z, Gao Z, Huang Y, Qi Y, Peng B, Wang H, Fu L, Song M, Chen P, Gao W, Ren B, Sun Y, Cai T, Feng X, Sui J, Li W (2012). Sodium taurocholate cotransporting polypeptide is a functional receptor for human hepatitis B and D virus. Elife.

[5] Watashi K, Urban S, Li W, Wakita T (2014). NTCP and beyond: opening the door to unveil hepatitis B virus entry. Int. J. Mol. Sci..

[6] Ni Y, Lempp FA, Mehrle S, Nkongolo S, Kaufman C, Fälth M, Stindt J, Königer C, Nassal M, Kubitz R, Sültmann H, Urban S (2014). Hepatitis B and D viruses exploit sodium taurocholate co-transporting polypeptide for species-specific entry into hepatocytes. Gastroenterology.

[7] Okuyama-Dobashi K, Kasai H, Tanaka T, Yamashita A, Yasumoto J, Chen W, Okamoto T, Maekawa S, Watashi K, Wakita T, Ryo A, Suzuki T, Matsuura Y, Enomoto N, Moriishi K (2015). Hepatitis B virus efficiently infects non-adherent hepatoma cells via human sodium taurocholate cotransporting polypeptide. Sci. Rep..

[8] Yan R, Zhang Y, Cai D, Liu Y, Cuconati A, Guo H (2015). Spinoculation enhances HBV infection in NTCP-reconstituted hepatocytes. PLoS ONE.

[9] Iwamoto M, Watashi K, Tsukuda S, Aly HH, Fukasawa M, Fujimoto A, Suzuki R, Aizaki H, Ito T, Koiwai O, Kusuhara H, Wakita T (2014). Evaluation and identification of hepatitis B virus entry inhibitors using HepG2 cells overexpressing a membrane transporter NTCP. Biochem. Biophys. Res. Commun..

[10] Kaneko M, Watashi K, Kamisuki S, Matsunaga H, Iwamoto M, Kawai F, Ohashi H, Tsukuda S, Shimura S, Suzuki R, Aizaki H, Sugiyama M, Park SY, Ito T, Ohtani N, Sugawara F, Tanaka Y, Mizokami M, Sureau C, Wakita T (2015). A novel tricyclic polyketide, Vanitaracin A, specifically inhibits the entry of Hepatitis B and D viruses by targeting sodium taurocholate cotransporting polypeptide. J. Virol..

[11] Tsukuda S, Watashi K, Hojima T, Isogawa M, Iwamoto M, Omagari K, Suzuki R, Aizaki H, Kojima S, Sugiyama M, Saito A, Tanaka Y, Mizokami M, Sureau C, Wakita T (2017). A new class of hepatitis B and D virus entry inhibitors, proanthocyanidin and its analogs, that directly act on the viral large surface proteins. Hepatology.

[12] Shimura S, Watashi K, Fukano K, Peel M, Sluder A, Kawai F, Iwamoto M, Tsukuda S, Takeuchi JS, Miyake T, Sugiyama M, Ogasawara Y, Park SY, Tanaka Y, Kusuhara H, Mizokami M, Sureau C, Wakita T (2017). Cyclosporin derivatives inhibit hepatitis B virus entry without interfering with NTCP transporter activity. J. Hepatol..

[13] Chen X, Cheung ST, So S, Fan ST, Barry C, Higgins J, Lai KM, Ji J, Dudoit S, Ng IO, Van De Rijn M, Botstein D, Brown PO (2002). Gene expression patterns in human liver cancers. Mol. Biol. Cell.

[14] Costantini S, Di Bernardo G, Cammarota M, Castello G, Colonna G (2013). Gene expression signature of human HepG2 cell line. Gene.

[15] Tnani M, Bayard BA (1999). Evidence for IRF-1-dependent gene expression deficiency in interferon unresponsive HepG2 cells. Biochim. Biophys. Acta.

[16] Li K, Chen Z, Kato N, Gale M, Lemon SM (2005). Distinct poly(I-C) and virus-activated signaling pathways leading to interferon-beta production in hepatocytes. J. Biol. Chem..

[17] Tsugawa Y, Kato H, Fujita T, Shimotohno K, Hijikata M (2014). Critical role of interferon-α constitutively produced in human hepatocytes in response to RNA virus infection. PLoS ONE.

[18] Dansako H, Ueda Y, Okumura N, Satoh S, Sugiyama M, Mizokami M, Ikeda M, Kato N (2016). The cyclic GMP-AMP synthetase-STING signaling pathway is required for both the innate immune response against HBV and the suppression of HBV assembly. FEBS J..

[19] Galle PR, Hagelstein J, Kommerell B, Volkmann M, Schranz P, Zentgraf H (1994). In vitro experimental infection of primary human hepatocytes with hepatitis B virus. Gastroenterology.

[20] Ishida Y, Yamasaki C, Yanagi A, Yoshizane Y, Fujikawa K, Watashi K, Abe H, Wakita T, Hayes CN, Chayama K, Tateno C (2015). Novel robust in vitro hepatitis B virus infection model using fresh human hepatocytes isolated from humanized mice. Am. J. Pathol..

[21] Huang HC, Chen CC, Chang WC, Tao MH, Huang C (2012). Entry of hepatitis B virus into immortalized human primary hepatocytes by clathrin-dependent endocytosis. J. Virol..

[22] Aly HH, Watashi K, Hijikata M, Kaneko H, Takada Y, Egawa H, Uemoto S, Shimotohno K (2007). Serum-derived hepatitis C virus infectivity in interferon regulatory factor-7-suppressed human primary hepatocytes. J. Hepatol..

[23] Decorsière A, Mueller H, van Breugel PC, Abdul F, Gerossier L, Beran RK, Livingston CM, Niu C, Fletcher SP, Hantz O, Strubin M (2016). Hepatitis B virus X protein identifies the Smc5/6 complex as a host restriction factor. Nature.

[24] Aly HH, Qi Y, Atsuzawa K, Usuda N, Takada Y, Mizokami M, Shimotohno K, Hijikata M (2009). Strain-dependent viral dynamics and virus-cell interactions in a novel in vitro system supporting the life cycle of blood-borne hepatitis C virus. Hepatology.

[25] Hong R, Bai W, Zhai J, Liu W, Li X, Zhang J, Cui X, Zhao X, Ye X, Deng Q, Tiollais P, Wen Y, Liu J, Xie Y (2013). Novel recombinant hepatitis B virus vectors efficiently deliver protein and RNA encoding genes into primary hepatocytes. J. Virol..

[26] Gamal W, Treskes P, Samuel K, Sullivan GJ, Siller R, Srsen V, Morgan K, Bryans A, Kozlowska A, Koulovasilopoulos A, Underwood I, Smith S, Del-Pozo J, Moss S, Thompson AI, Henderson NC, Hayes PC, Plevris JN, Bagnaninchi PO, Nelson LJ (2017). Low-dose acetaminophen induces early disruption of cell-cell tight junctions in human hepatic cells and mouse liver. Sci. Rep..

[27] Aly HH, Shimotohno K, Hijikata M (2009). 3D cultured immortalized human hepatocytes useful to develop drugs for blood-borne HCV. Biochem. Biophys. Res. Commun..

[28] Moolla N, Kew M, Arbuthnot P (2002). Regulatory elements of hepatitis B virus transcription. J. Viral Hepat..

[29] Okamura H, Nio Y, Akahori Y, Kim S, Watashi K, Wakita T, Hijikata M (2016). Fatty acid biosynthesis is involved in the production of hepatitis B virus particles. Biochem. Biophys. Res. Commun..

[30] Yan R, Zhao X, Cai D, Liu Y, Block TM, Guo JT, Guo H (2015). The interferon-inducible protein tetherin inhibits hepatitis B virus virion secretion. J. Virol..

[31] Sells MA, Chen ML, Acs G (1987). Production of hepatitis B virus particles in Hep G2 cells transfected with cloned hepatitis B virus DNA. Proc. Natl. Acad. Sci. U.S.A..

[32] Gripon P, Rumin S, Urban S, Le Seyec J, Glaise D, Cannie I, Guyomard C, Lucas J, Trepo C, Guguen-Guillouzo C (2002). Infection of a human hepatoma cell line by hepatitis B virus. Proc. Natl. Acad. Sci. U.S.A..

[33] Shlomai A, Schwartz RE, Ramanan V, Bhatta A, de Jong YP, Bhatia SN, Rice CM (2014). Modeling host interactions with hepatitis B virus using primary and induced pluripotent stem cell-derived hepatocellular systems. Proc. Natl. Acad. Sci. U.S.A..

[34] Kaneko S, Kakinuma S, Asahina Y, Kamiya A, Miyoshi M, Tsunoda T, Nitta S, Asano Y, Nagata H, Otani S, Kawai-Kitahata F, Murakawa M, Itsui Y, Nakagawa M, Azuma S, Nakauchi H, Nishitsuji H, Ujino S, Shimotohno K, Iwamoto M, Watashi K, Wakita T, Watanabe M (2016). Human induced pluripotent stem cell-derived hepatic cell lines as a new model for host interaction with hepatitis B virus. Sci. Rep..

[35] Sakurai F, Mitani S, Yamamoto T, Takayama K, Tachibana M, Watashi K, Wakita T, Iijima S, Tanaka Y, Mizuguchi H (2017). Human induced-pluripotent stem cell-derived hepatocyte-like cells as an in vitro model of human hepatitis B virus infection. Sci. Rep..

[36] Ko C, Chakraborty A, Chou WM, Hasreiter J, Wettengel JM, Stadler D, Bester R, Asen T, Zhang K, Wisskirchen K, McKeating JA, Ryu WS, Protzer U (2018). Hepatitis B virus genome recycleing and de novo secondary infection events maintain stable cccDNA levels. J. Hepatol..

[37] König A, Yang J, Jo E, Ho K, Park P, Kim H, Than TT, Song X, Qi X, Dai X, Park S, Shum D, Ryu RS, Kim JH, Yoon SK, Park JY, Ahn SH, Han KH, Gerlich WH, Windisch MP (2019). Efficient long-term amplification of hepatitis B virus isolates after infection of slow proliferating HepG2-NTCP cells. J. Hepatol..

[38] Li J, Xu Z, Zheng Y, Johnson DL, Ou JH (2002). Regulation of hepatocyte nuclear factor 1 activity by wild-type and mutant hepatitis B virus X proteins. J. Virol..

[39] Zheng Y, Li J, Ou JH (2004). Regulation of hepatitis B virus core promoter by transcription factors HNF1 and HNF4 and the viral X protein. J. Virol..

[40] Lazar C, Durantel D, Macovei A, Zitzmann N, Zoulim F, Dwek RA, Branza-Nichita N (2007). Treatment of hepatitis B virus-infected cells with alpha-glucosidase inhibitors results in production of virions with altered molecular composition and infectivity. Antiviral Res..

[41] Lambert C, Döring T, Prange R (2007). Hepatitis B virus maturation is sensitive to functional inhibition of ESCRT-III, Vps4, and gamma 2-adaptin. J. Virol..

[42] Kian Chua P, Lin MH, Shih C (2006). Potent inhibition of human hepatitis B virus replication by a host factor Vps4. Virology.

[43] Yao WL, Ikeda S, Tsukamoto Y, Shindo K, Otakaki Y, Qin M, Iwasawa Y, Takeuchi F, Kaname Y, Chou YC, Chang C, Watashi K, Wakita T, Noda T, Kato H, Fujita T (2017). Establishment of a human hepatocellular cell line capable of maintaining long-term replication of hepatitis B virus. Int. Immunol..

[44] Inamura K, Emoto K, Ichihara H, Sasaki K, Iwasa T, Kojima R, Kawabe M, Komizu Y, Matsumoto Y, Matsushita T (2018). Evaluation of an in vitro approach to the prediction of in vivo effects on multidrug resistance in human hepatoma cells. J. Carcinog Mutagen..

[45] Ogura N, Watashi K, Noguchi T, Wakita T (2014). Formation of covalently closed circular DNA in Hep38.7-Tet cells, a tetracycline inducible hepatitis B virus expression cell line. Biochem. Biophys. Res. Commun..

[46] Kushima Y, Wakita T, Hijikata M (2010). A disulfide-bonded dimer of the core protein of hepatitis C virus is important for virus-like particle production. J. Virol..

[47] Miyanari Y, Atsuzawa K, Usuda N, Watashi K, Hishiki T, Zayas M, Bartenschlager R, Wakita T, Hijikata M, Shimotohno K (2007). The lipid droplet is an important organelle for hepatitis C virus production. Nat. Cell Biol..

[48] Sugiyama M, Tanaka Y, Kato T, Orito E, Ito K, Acharya SK, Gish RG, Kramvis A, Shimada T, Izumi N, Kaito M, Miyakawa Y, Mizokami M (2006). Influence of hepatitis B virus genotypes on the intra- and extracellular expression of viral DNA and antigens. Hepatology.

