# Supplementary material for: Establishment of a novel hepatitis B virus culture system using immortalized human hepatocytes
Source: Sci Rep. 2020 Dec 10;10:21718. doi: 10.1038/s41598-020-78655-x (PMC7729873; doi:10.1038/s41598-020-78655-x)
Supplement: Supplementary file 1 — Supplementary Information. [file 41598_2020_78655_MOESM1_ESM.pdf]

Supplementary Information

Establishment of a novel hepatitis B virus culture system using immortalized human hepatocytes

Yuichi Akahori [1, 2], Hiroki Kato [1, 2], Takashi Fujita [1, 2], Kohji Moriishi [3], Yasuhito Tanaka [4], Koichi Watashi [5], Michio Imamura[6], Kazuaki Chayama[6], Takaji Wakita [5], Makoto Hijikata [1, 2, *]

1. Institute for Frontier Life and Medical Sciences, Kyoto University, Japan;
2. Graduate School of Biostudies, Kyoto University, Japan;
3. Interdisciplinary Graduate School of Medicine and Engineering, University of Yamanashi, Japan;
4. Graduate School of Medical Sciences, Nagoya City University, Japan;
5. Department of Virology II, National Institute of Infectious Diseases, Japan;
6. Department of Gastroenterology and Metabolism, Applied Life Sciences, Institute of Biomedical and Health Sciences, Hiroshima University, Japan;

* corresponding author mhijikat@infront.kyoto-u.ac.jp

Supplemental materials and method

Detection of HBV cccDNA by nested-PCR

Total cellular DNA was isolated from the Cellbed-cultured cells using phenol-chloroform-isoamyl alcohol (25:24:1) (Nacalai, Kyoto, Japan) and treated with T5 exonuclease (New England Biolabs, MA, USA), as described previously [49]. Detection of T5 exonuclease-resistant HBV cccDNA by nested-PCR using TaKaRa Taq polymerase (Takara, Osaka, Japan) and specific primer set (listed in Supplemental Table 1). The 1st PCR products were purified using DNeasy Blood and Tissue kit (Qiagen, Hilden, Germany), and then the eluted samples were used as a template of the 2nd PCR reaction.

Supplemental Table 1. The sequence of primer sets for nested-PCR

1st PCR	F	5’-CCGTGTGCACTTCGCTTCA-3’
	R	5’-GCACAGCTTGGAGGCTTGA-3’
2 nd PCR	F	5’-TGAACGCCCACCAATTCTTG-3’
	R	5’-GAGATGATTAGGCAGAGGTG-3’

Supplemental reference

[49] **Qu B**, Ni Y, Lempp FA, Vondran FWR, Urban S. T5 Exonuclease hydrolysis of hepatitis B virus replicative intermediates allows reliable quantification and fast drug efficacy testing of covalently closed circular DNA by PCR. J Virol. 2018 Dec 1; 92(23): e01117-18.

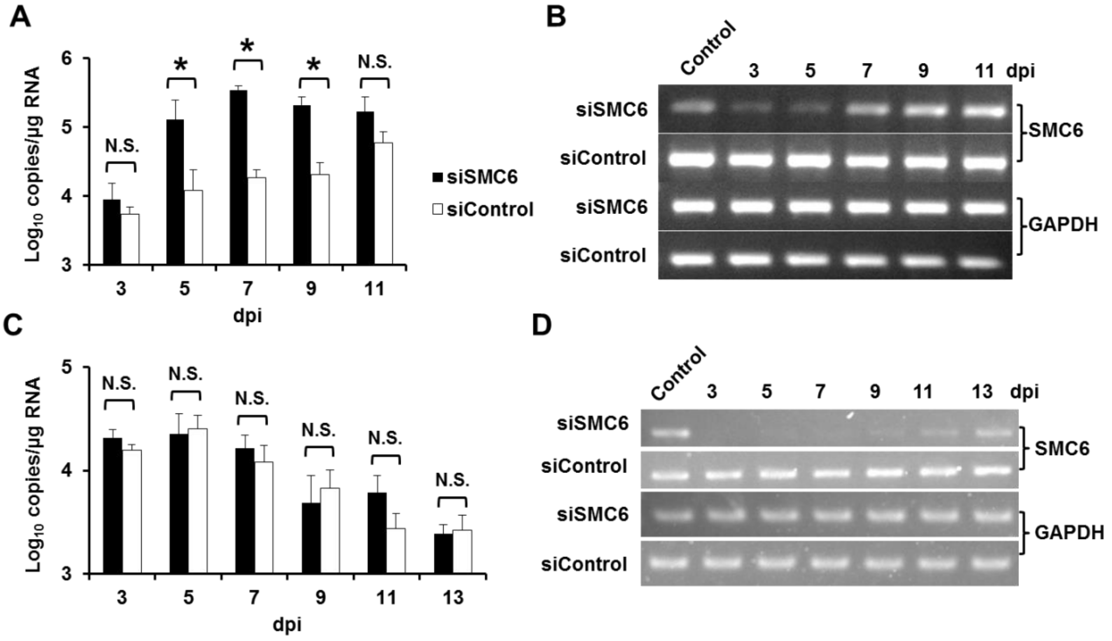

Supplemental Figure S1. (A) HepG2-hNTCP-C4 cells were transfected with SMC6 siRNA (siSMC6, shown in black bar) or control siRNA (siControl, shown in white bar). After HBVcc infection, the copy number of HBV pgRNA in the cells harvested at indicated time points was evaluated by qRT-PCR. (B) The mRNAs of SMC6 and GAPDH in HepG2-hNTCP-C4 cells transfected with siSMC6 or siControl were detected by RT-PCR. GAPDH was used as an internal control. Full-length gel images were presented in Supplement Fig. S16. (C) E/NtG8 cells were transfected with siSMC6 or siControl. After HBVcc infection, the copy number of HBV pgRNA in the cells was evaluated by qRT-PCR. (D) The mRNAs of SMC6 and GAPDH in E/NtG8 cells transfected with siSMC6 or siControl were detected by RT-PCR as panel B. Full-length gel images were presented in Supplement Fig. S17 and S18. Levels of significance: * and N. S. indicate “$p < 0.05$” and “no significant difference”, respectively.

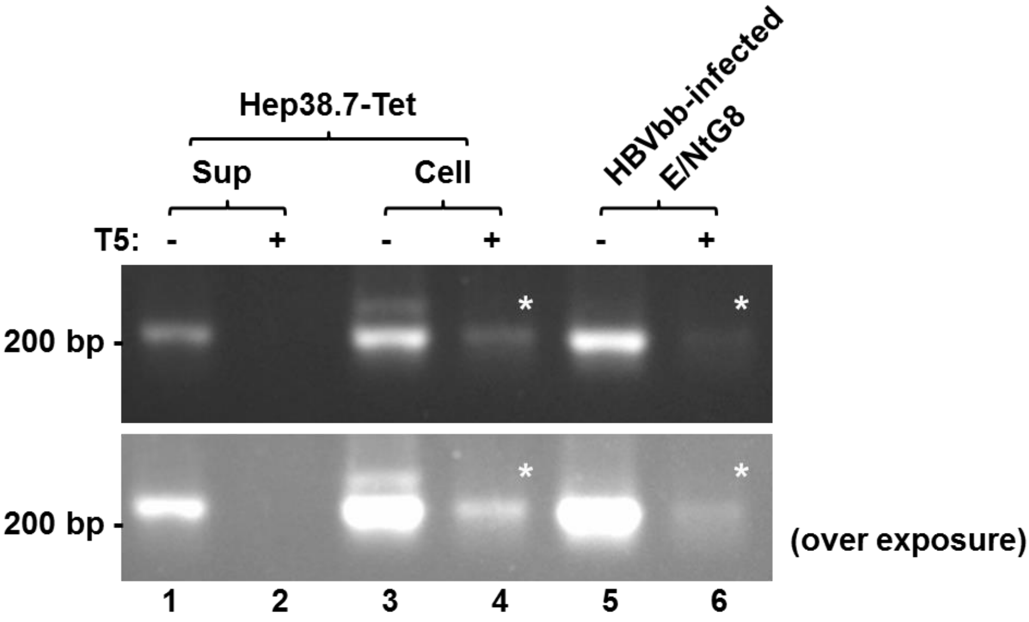

Supplemental Figure S2. Detection of HBV cccDNA in HBVbb-infected E/NtG8 cells cultured on Cellbed. As positive control of the detection of cccDNA, Hep38.7-Tet cells were cultured with tetracycline-containing medium overnight. After removal of tetracycline, the cells were cultured for additional 8 days and total cellular DNA was purified (denoted Cell). At the same time, extracellular HBV DNA included in HBV particles was isolated from the culture medium of Hep38.7-Tet cells (denoted Sup). Total cellular DNA of cellbed-cultured E/NtG8 cells infected with HBVbb genotype C, as in Fig. 3A, was purified. The cccDNA of HBV in those DNA samples was detected by nested PCR after the treatment with (+) or without (-) T5 exonuclease (denoted T5). The presence of T5 exonuclease resistant HBV DNA, that is HBV cccDNA, is indicated by asterisk (lanes 4 and 6), while the relaxed circular HBV DNA included in HBV particles was no more resistant to T5 exonuclease treatment (no band in lane 2). The lower panel shows the overexposed image of upper panel. Full-length gel images were presented in Supplement Fig. S21 and S22.

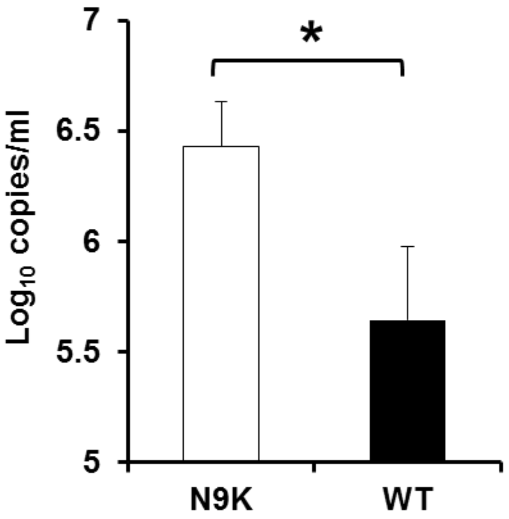

Supplemental Figure S3. HBVbb (genotype A) infection experiment using E/NtG8 cells cultured on Cellbed. 3D-cultured E/NtG8 cells were infected with HBVbb in the presence of myr-47N9K (N9K) (white bar) or myr-47WT (WT) (black bar). The amount of extracellular HBV DNA in the culture media was evaluated by qPCR at 15 dpi. Levels of significance: * indicates $p < 0.05$.

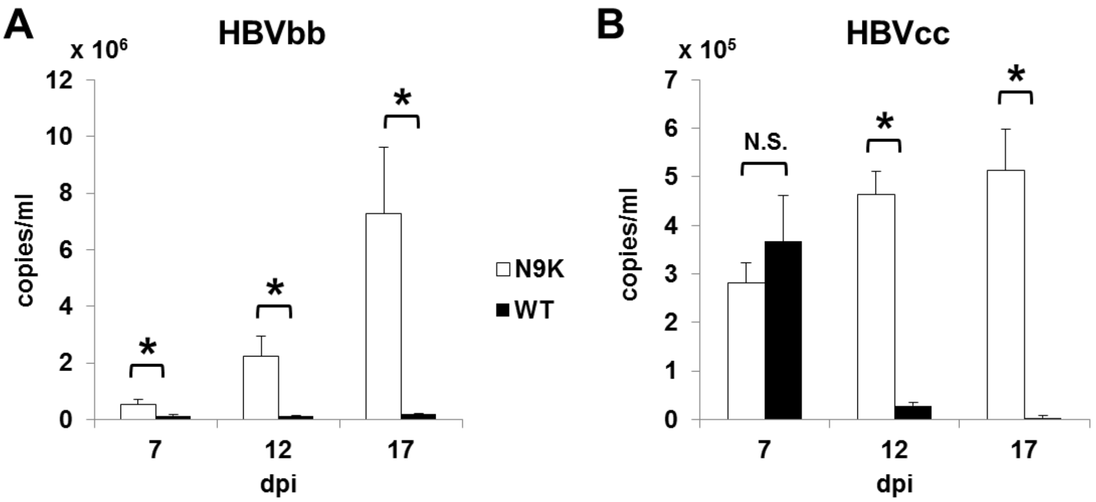

Supplemental Figure S4. Comparison of the infectivity between HBVbb (A) and HBVcc (B) using PXB-cells. Two days after infection of HBVbb or HBVcc, the PXB-cells were cultured for additional 15 days, as described previously [20] in the presence of myr-47WT (WT) (black bar) or myr-47N9K (N9K) (white bar). The amounts of HBV DNA in each culture medium were evaluated by qPCR. Data of the cells treated with myr-47WT (WT) or myr-47N9K (N9K) during the inoculation. Levels of significance: * and N. S. indicate “$p < 0.05$” and “no significant difference”, respectively.

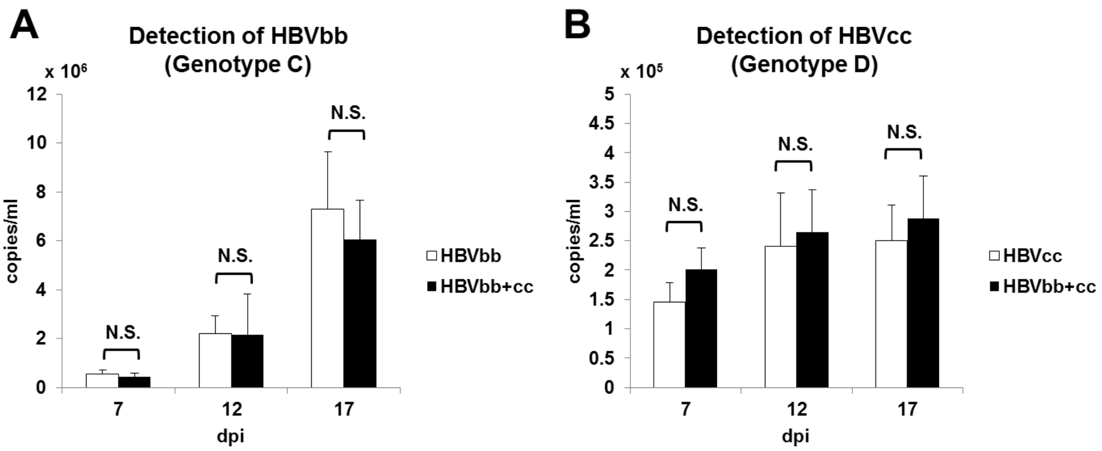

Supplemental Figure S5. Permissiveness of PXB-cells HBVbb derived from HBV genotype C in the presence of HBVcc derived from HBV genotype D. (A) The amounts of HBV DNA in each culture medium were evaluated by qPCR. The data of single HBV infection, shown in the white bar, is same results as in Fig. 7A. (B) The amounts of HBV DNA in each culture medium were evaluated by qPCR. The data of single HBV infection, shown in the white bar, is same sample as in Fig. 7B. Levels of significance: * and N. S. indicate “$p < 0.05$” and “no significant difference”, respectively.

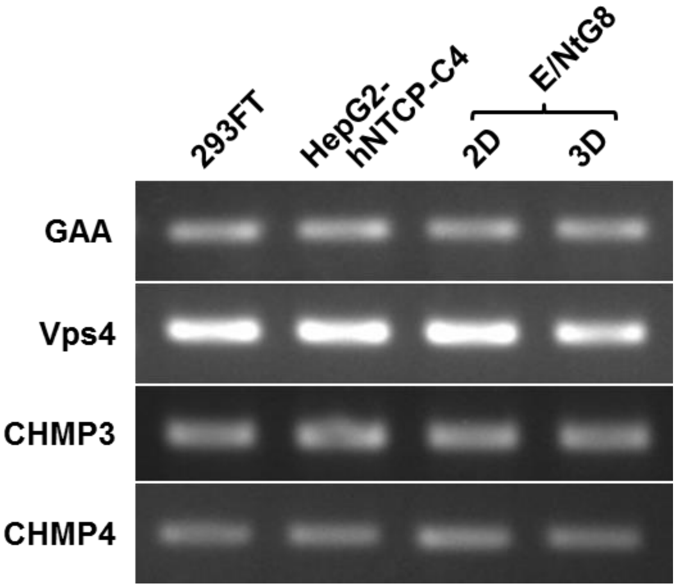

Supplemental Figure S6. The expression of HBV egression-related host genes, α-glycosidase (GAA) ([35] Lazar C. 2007), Vps4 ([36] Lambert C. 2007), charged multivesicular body protein 3 (CHMP3) and CHMP4 ([37] Kian Chua P. 2006) in 293FT, HepG2-hNTCP-C4, 2D-cultured (2D) and 3D-cultured E/NtG8 cells (3D) were evaluated by RT-PCR. RT-PCR was performed on the same sample as in Fig. 5C. Full-length gel images were presented in Supplement Fig. S19 and S20.

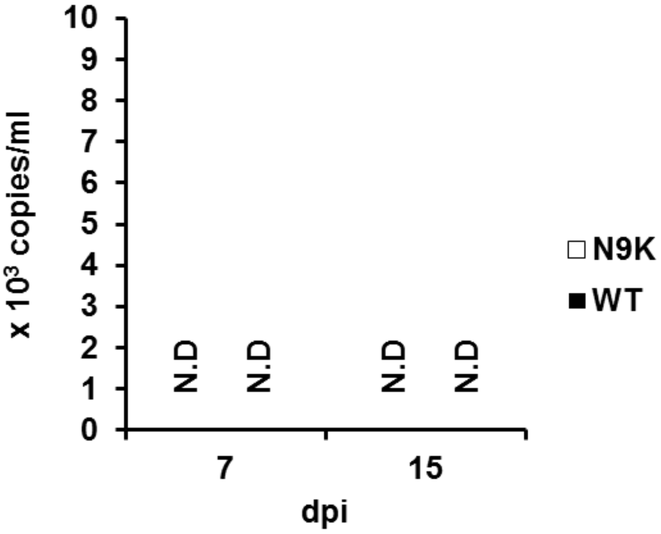

Supplemental Figure S7. HBVbb infection experiment using 2D-cultured HepG2-hNTCP-C4 cells. HepG2-hNTCP-C4 cells were pretreated with 1 μM myr-47WT or myr-47N9K for 3 h prior to and during HBV infection. After HBVbb infection, culture media of the HepG2-hNTCP-C4 cells were collected every 2 days. The amounts of extracellular HBV DNA in the media were evaluated by qPCR at 15 dpi. N. D. indicates “not detectable”.

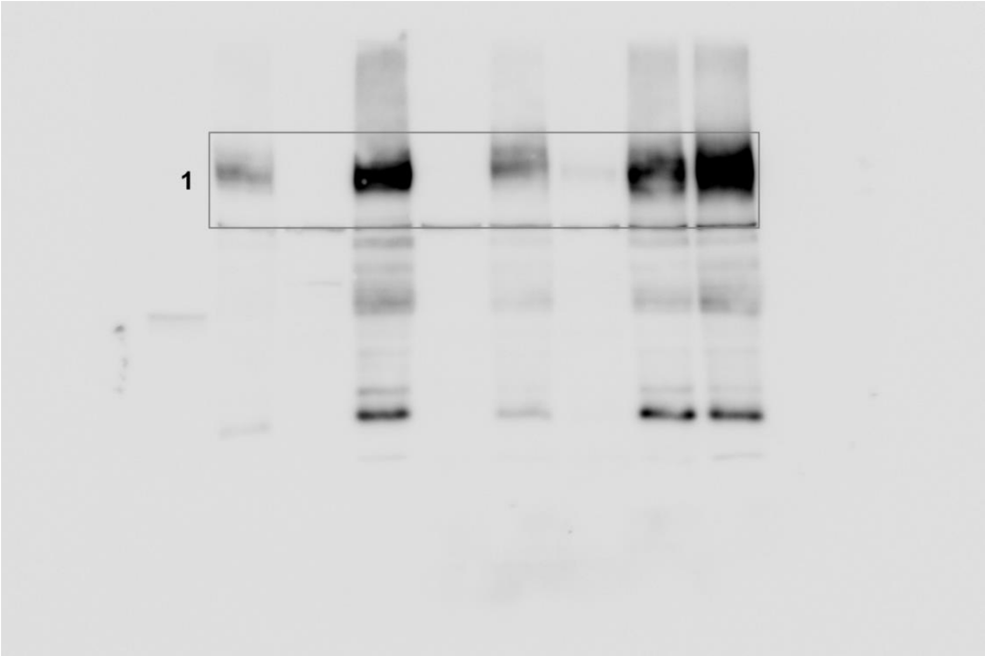

Supplemental Figure S8. Full-length blot image before cropping for Figure 1A.
The expression of NTCP-tGFP (1) was shown.

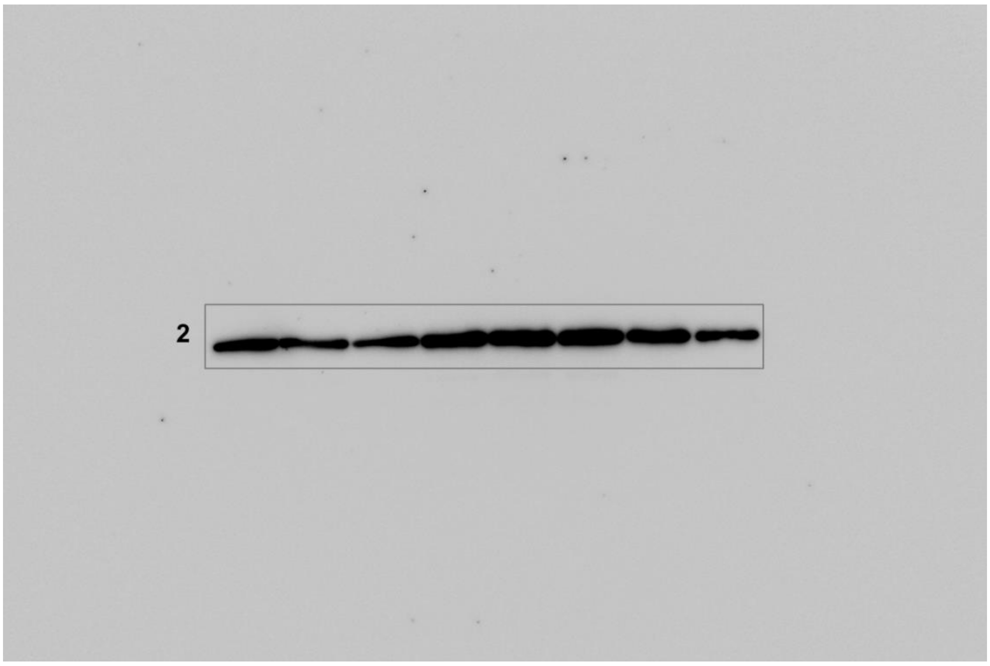

Supplemental Figure S9. Full-length blot image before cropping for Figure 1A.
The expression of GAPDH (2) was shown.

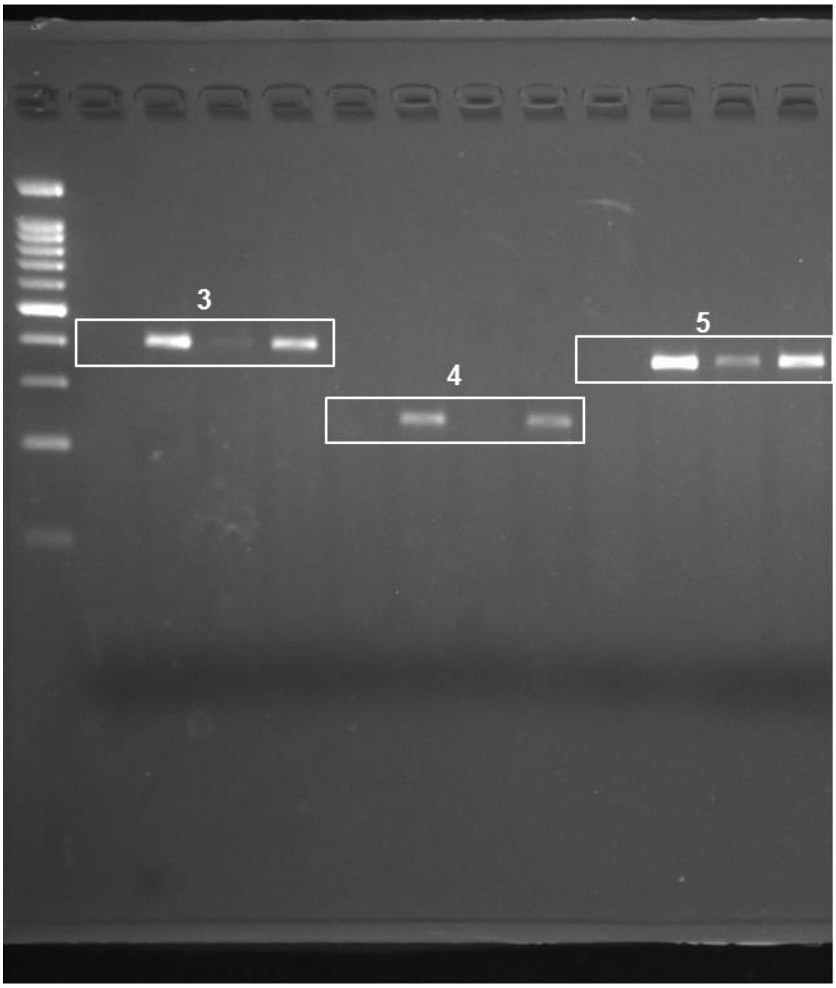

Supplemental Figure S10. Full-length gel image before cropping for Figure 6C.
The expression of ALB (3), HNF-1α (4) and HNF-4α (5) were shown.

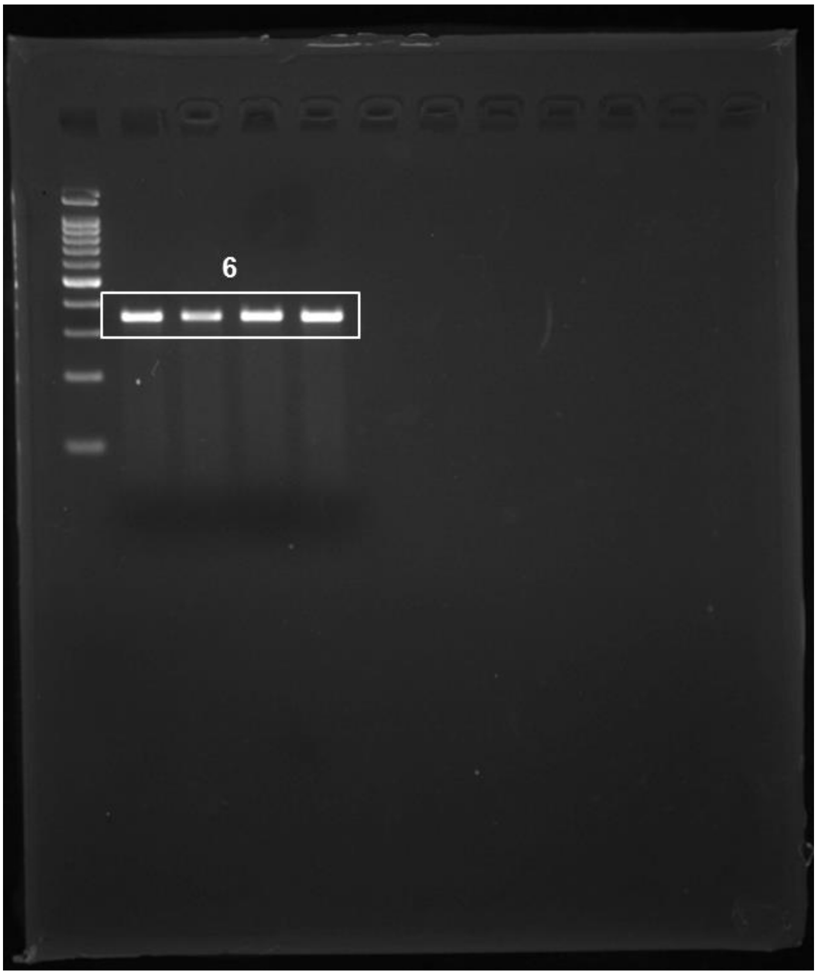

Supplemental Figure S11. Full-length gel image before cropping for Figure 6C.
The expression of ACC1 (6) expression was shown.

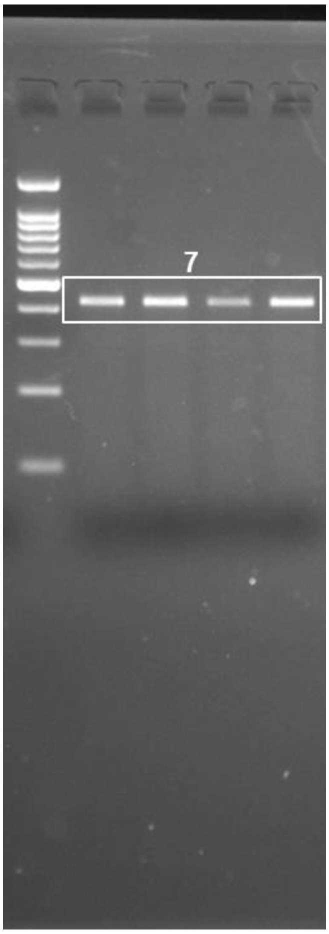

Supplemental Figure S12. Full-length gel image before cropping for Figure 6C.
The expression of FAS (7) expression was shown.

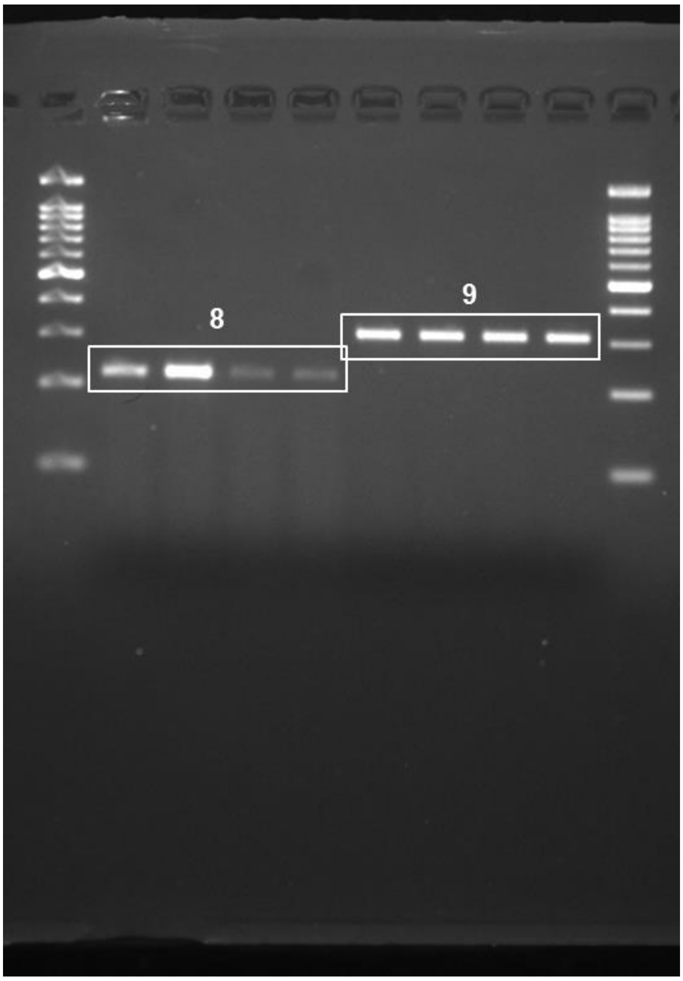

Supplemental Figure S13. Full-length gel image before cropping for Figure 6C.
The expression of BST2 (8) and GAPDH (9) were shown.

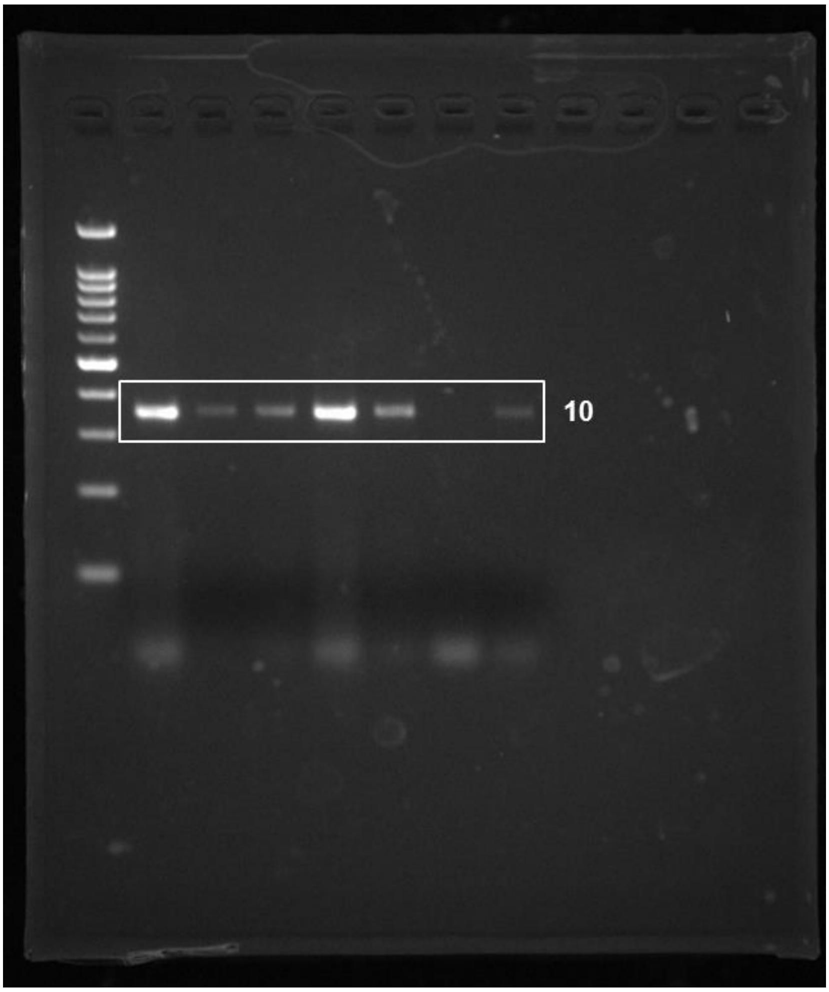

Supplemental Figure S14. Full-length gel image before cropping for Figure 6D.

The expression of SMC6 (10) was shown.

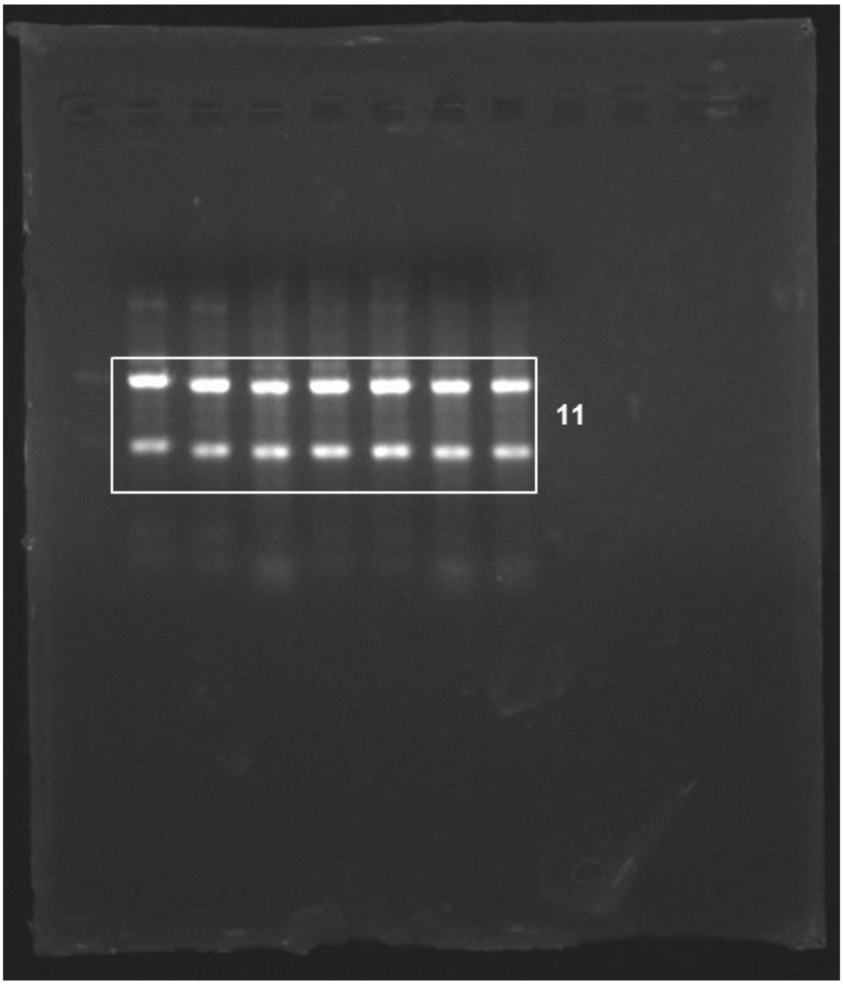

Supplemental Figure S15. Full-length gel image before cropping for Figure 6D.
The expression of rRNA (11) was shown.

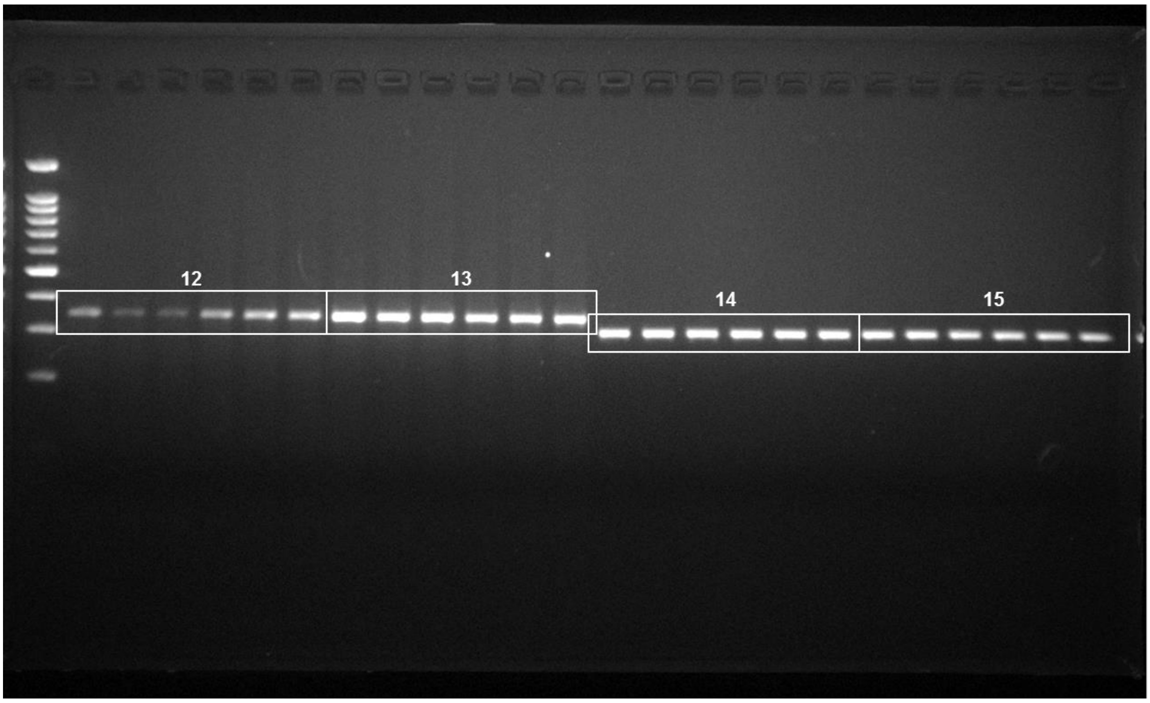

Supplemental Figure S16. Full-length gel image before cropping for Figure S1B.

The expression of SMC6 in SMC6 siRNA-transfected HepG2-hNTCP-C4 cells (12) and control siRNA-transfected HepG2-hNTCP-C4 cells (13). And the expression of GAPDH in SMC6 siRNA-transfected HepG2-hNTCP-C4 cells (14) and control siRNA-transfected HepG2-hNTCP-C4 cells (15).

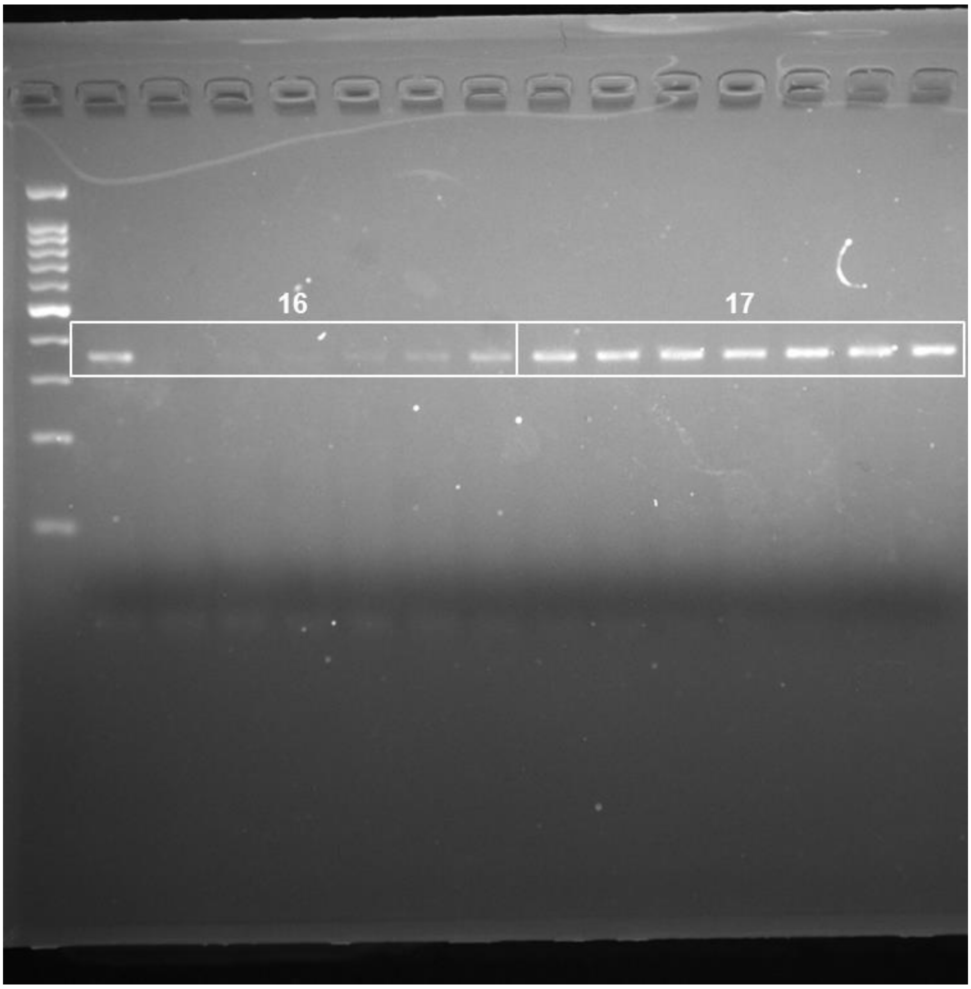

Supplemental Figure S17. Full-length gel image before cropping for Figure S1D.

The expression of SMC6 in SMC6 siRNA-transfected E/NtG8 cells (16) and control siRNA-transfected E/NtG8 cells (17).

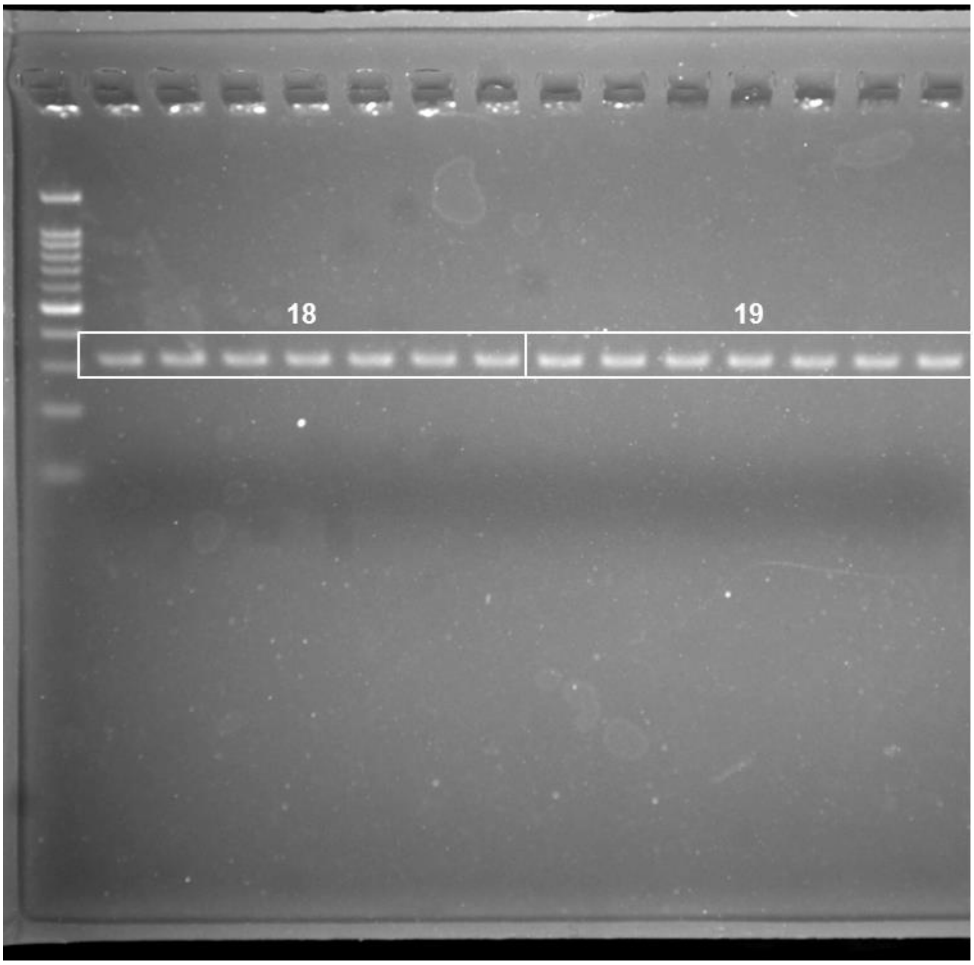

Supplemental Figure S18. Full-length gel image before cropping for Figure S1D.

The expression of GAPDH in SMC6 siRNA-transfected E/NtG8 cells (18) and control siRNA-transfected E/NtG8 cells (19).

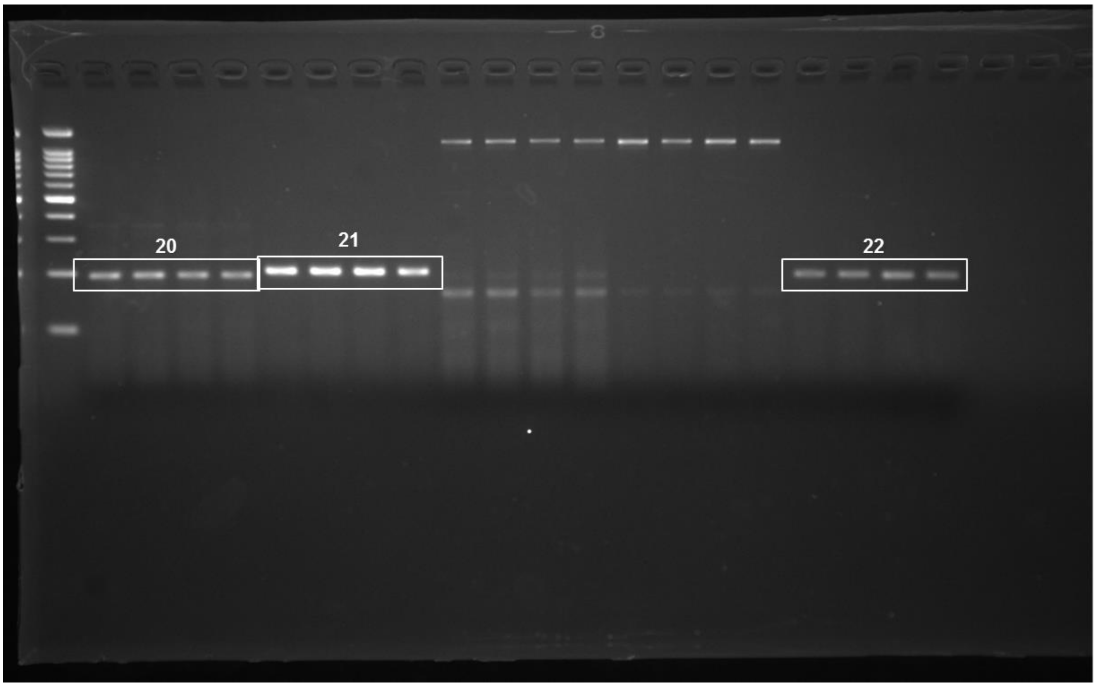

Supplemental Figure S19. Full-length gel image before cropping for Figure S4.
The expression of GAA (20), Vsp4 (21) and CHMP4 (22) were shown.

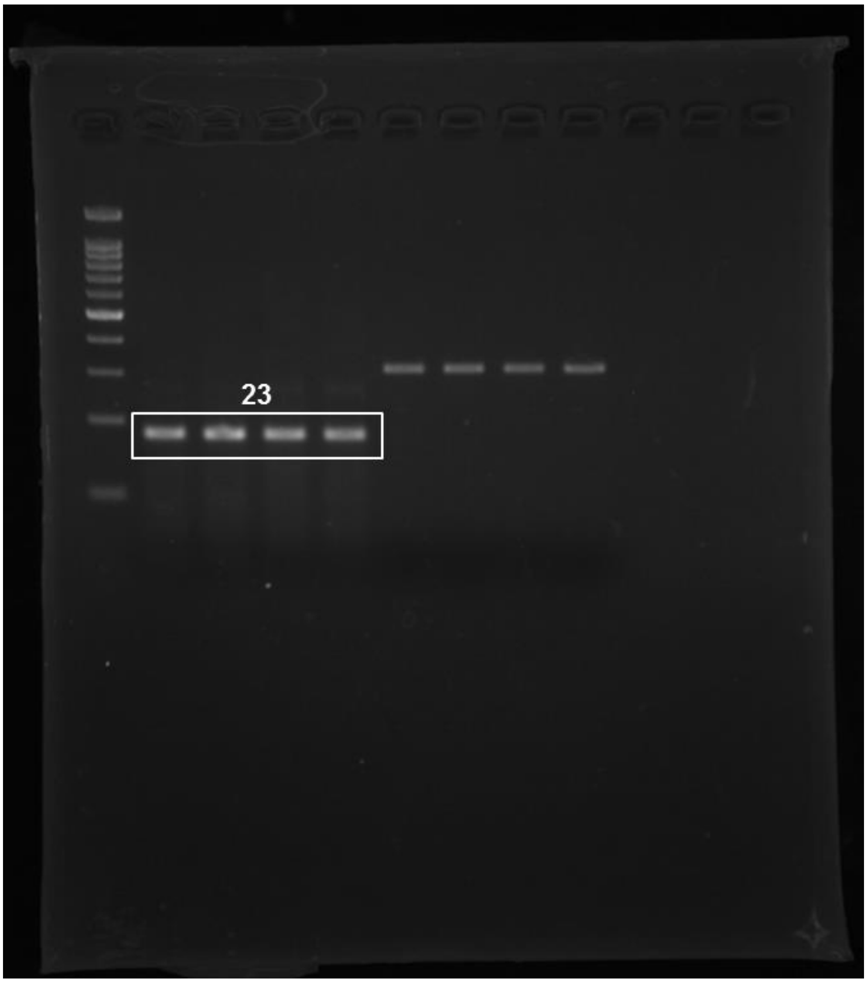

Supplemental Figure S20. Full-length gel image before cropping for Figure S4.
The expression of CHMP3 (23) was shown.

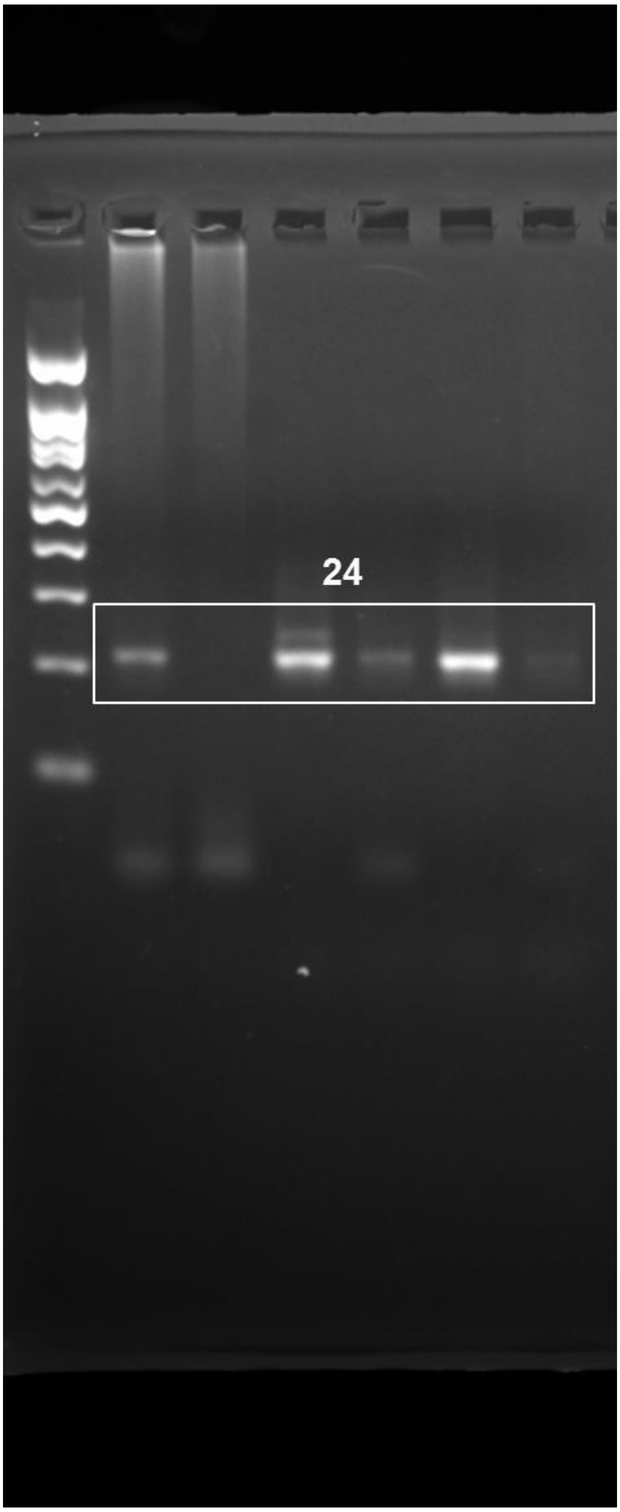

Supplemental Figure S21. Full-length gel image before cropping for Figure S2.
The products of the 2nd PCR (24) were shown.

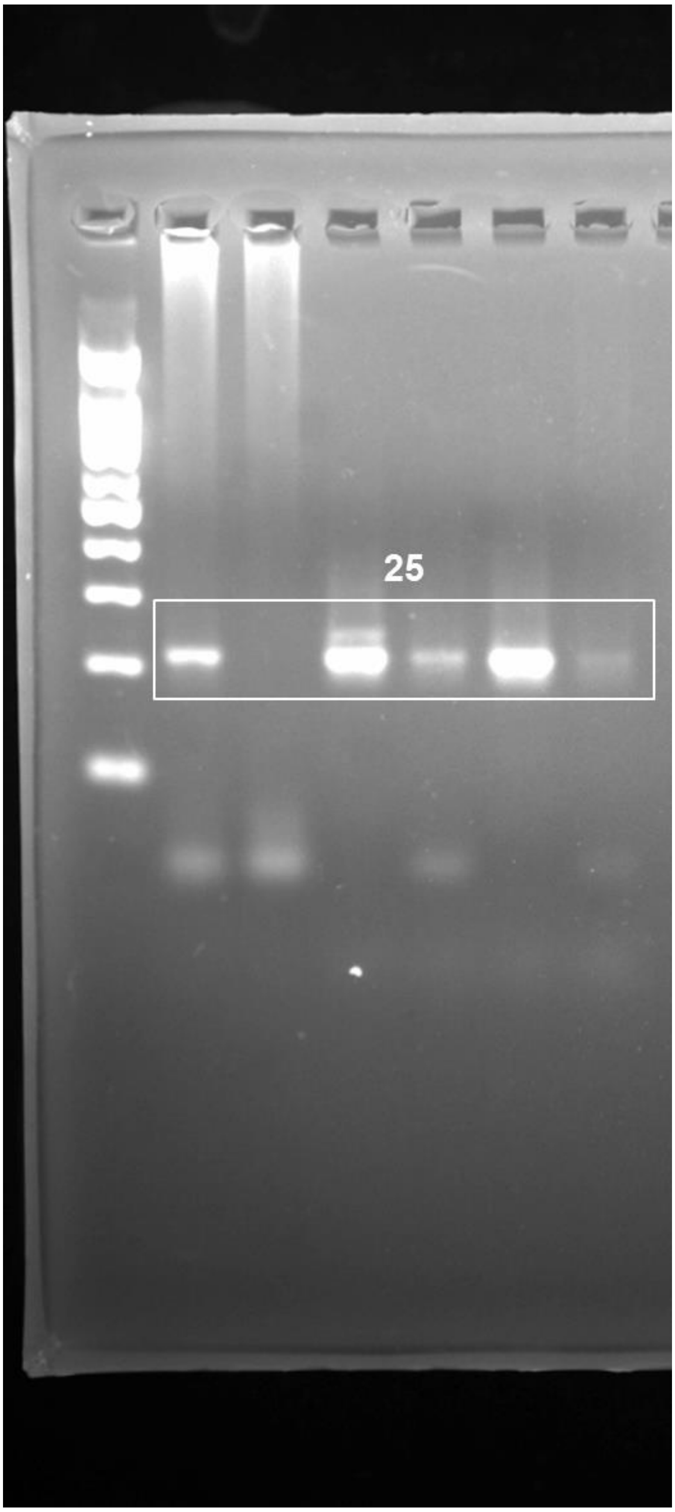

Supplemental Figure S22. Full-length gel image before cropping for Figure S2.

The over exposure image of Figure S21 was shown.
